# Spreading of the High-Pathogenicity Avian Influenza (H5N1) Virus of Clade 2.3.4.4b into Uruguay

**DOI:** 10.3390/v15091906

**Published:** 2023-09-11

**Authors:** Ana Marandino, Gonzalo Tomás, Yanina Panzera, Carmen Leizagoyen, Ramiro Pérez, Lucía Bassetti, Raúl Negro, Sirley Rodríguez, Ruben Pérez

**Affiliations:** 1 Sección Genética Evolutiva, Departamento de Biología Animal, Instituto de Biología, Facultad de Ciencias, Universidad de la República, Iguá 4225, Montevideo 11400, Uruguay; amarandino@fcien.edu.uy (A.M.); gtomas@fcien.edu.uy (G.T.); ypanzera@fcien.edu.uy (Y.P.); 2 Dirección Nacional de Biodiversidad y Servicios Ecosistémicos (DINABISE), Ministerio de Ambiente, Juncal 1385, Montevideo 11100, Uruguay; carmenleizagoyen@ambiente.gub.uy; 3 Departamento de Virología, División de Laboratorios Veterinarios “Miguel C. Rubino”, Dirección General de Servicios Ganaderos, Ministerio de Ganadería, Agricultura y Pesca, Ruta 8 “Brigadier Gral. Juan A. Lavalleja” Km 17,000, Montevideo 12100, Uruguay; raperez@mgap.gub.uy (R.P.); lbassetti@mgap.gub.uy (L.B.); rnegro@mgap.gub.uy (R.N.)

**Keywords:** AIV, South America, genetic lineage, backyard poultry, wildlife

## Abstract

Background: Avian influenza viruses (genus *Alphainfluenzavirus*, family *Orthomyxoviridae*) infect avian and mammal hosts. In 2022, the high pathogenicity avian influenza virus (H5N1) spread to South America, resulting in the loss of thousands of wild birds, including endangered species, and severely impacting the global poultry industry. Objectives: We analyzed the complete genomes of influenza viruses obtained from wild birds and backyard poultry in Uruguay between February and May 2023. Methods: Twelve complete genomes were obtained in 2023 from cloacal swabs using Illumina sequencing. Genomes were phylogenetically analyzed with regional and global strains. Findings: The identified strains have multiple basic amino acids at the hemagglutinin cleavage sites, which is typical for highly pathogenic strains. The Uruguayan viruses belonged to hemagglutinin clade 2.3.4.4b of the H5N1 subtype. A reassortment in North America has resulted in some segments of South American strains being of Eurasian or North American origins. The Uruguayan viruses shared a common ancestor with South American strains from Argentina and Chile. The influenza viruses displayed a spatiotemporal divergence pattern rather than being host-specific. Main Conclusions: The arrival of the 2.3.4.4b clade in Uruguay may have been mediated by birds that acquired the virus from Argentine and Chilean waterfowl migrating in the Pacific Flyway.

## 1. Introduction

Avian influenza viruses (AIV) belong to the *Alphainfluenzavirus influenzae* species of the *Orthomyxoviridae* family [1]. AIV infects avian and mammal hosts, but birds are widely believed to be the ancestral hosts [2]. The virus has a negative-sense single-stranded RNA consisting of eight genomic segments varying in size from 890 to 2341 nucleotides. Segments 1, 2, and 3 encode three viral polymerase subunits (PB1, PB2, and PA). Segments 4 and 6 encode the haemagglutinin (HA) and the neuraminidase (NA). Segment 5 encodes the nucleoprotein (NP), while segment 7 encodes the matrix (M) protein. Segment 8 encodes the non-structural proteins (NS). Bird viruses have sixteen different subtypes of haemagglutinin (H1-H16) and nine subtypes of NA (N1-N9) [3].

Like other RNA viruses, AIV mutates rapidly, usually resulting in two to eight substitutions per 1000 sites per year [4]. Viral segmentation further boosts its evolutionary speed by allowing reassortment events between two viruses infecting the same host cell. 

In wild birds, AIV infections are usually asymptomatic or of low pathogenicity [5,6]. The viral replication occurs primarily in the gastrointestinal tract and results in fecal shedding with little oropharyngeal shedding [7]. A high pathogenicity avian influenza pathotype in chickens causes severe damage with high mortality, requiring immediate notification to health authorities. High-pathogenicity avian influenza virus (HPAIV) strains are primarily characterized by multiple basic amino acid residues at the HA cleavage site that can be processed by ubiquitous proteases, resulting in a widespread disease with increased pathogenicity. These strains are usually of the H5 or H7 subtypes, but not all H5 and H7 viruses have high pathogenicity.

All circulating HPAIV strains of the H5 subtype are descended from a common ancestor that emerged in Southern China in the 1990s. The A/goose/Guangdong/1/96 H5N1 strain isolated from farmed geese in Sanshui, a small rural town in Guangdong Province, is considered the representative ancestor, and its descendant formed the Gs/GD lineage [8,9]. Since its first detection in 1996, this lineage has undergone a reassortment of internal protein-coding segments and diversification into multiple phylogenetic groups or clades [10,11,12,13]. However, the lineage retains the same HA segment (H5) and is further differentiated phylogenetically into second-, third-, and fourth-order HA clades. From 2014 onward, clade 2.3.4.4 has become more prevalent and expanded into eight new clades named with a fifth-order letter ranging from “a” to “h”. The H5 viruses carrying the HA gene of clade 2.3.4.4b were particularly successful and expanded their geographical and host ranges. At the beginning of 2020, viruses bearing the clade 2.3.4.4b HA gene infected domestic poultry and wild birds, leading to the loss of millions of domestic birds in Europe, Africa, and Asia [14]. Moreover, these H5 viruses underwent reassortment with other avian influenza viruses and acquired N1-N6 segments. This group of viruses is collectively known as H5Nx [14,15]. 

During 2020, H5N1 AIV arose from previously circulating 2.3.4.4b H5Nx viruses and spread predominantly via migratory birds. The H5N1 viruses bearing the clade 2.3.4.4b HA have almost entirely replaced the other H5Nx types since they emerged in October 2020 in the Netherlands [16]. The high-pathogenicity avian influenza (H5N1) virus of clade 2.3.4.4b has caused devasting outbreaks that have caused the deaths of numerous wild and domestic birds in Asia, Europe, Africa, and America [17,18,19,20,21,22]. In 2023, HPAIV spread to South America, resulting in the loss of thousands of wild birds, including endangered species, and severely impacting the global poultry industry [23,24]. Detecting human cases has also raised significant public health concerns [19]. 

In the current scenario, it is highly relevant to understand the evolution and emergence of these potentially pandemic AIV strains. This study examined the entire genomes of AIV strains found in wild birds and backyard poultry in Uruguay. We aimed to gain insights into the genetic diversity and transmission patterns of AIV in South America.

## 2. Materials and Methods

### 2.1. Ethics Statements 

This study did not require ethical approval since the activities were conducted as part of routine animal surveillance and public health response to avian influenza outbreaks.

### 2.2. Sample Collections

Samples were collected from wild birds and backyard poultry from AIV outbreaks detected in different regions of Uruguay between February and May 2023 in Uruguay (Table 1). Orotracheal or cloacal swabs were obtained from two wild black-necked swans (*Cygnus melancoryphus*) and ten backyard poultry (Table 1). Each swab was placed in a 5 mL sterile tube containing 3 mL of Dulbecco’s modified Eagle medium (Gibco, Waltham, MA, USA) containing 1% bovine adult serum and 2% antibiotic/antimycotic solution (Sigma-Aldrich, St. Louis, MO, USA).

### 2.3. AIV Detection

RNA extraction was performed using the TACO™ mini Automatic Nucleic Acid Extraction System (GeneReach Biotechnology, Taiwan, China). Reverse transcription-quantitative real-time polymerase chain reactions (RT-qPCR) were performed using the VetMax Gold AIV Detection Kit (Thermo Fisher, Waltham, MA, USA). This kit targets the matrix and nucleoprotein coding sequences. Amplification reactions were performed on 7500 Real-Time PCR Systems (Applied Biosystems, Waltham, MA, USA).

### 2.4. HA Subtyping

Detection of Eurasian lineage influenza A subtype H5 was performed using an RT-qPCR protocol adapted from a National Veterinary Services Laboratories (NVSL) United States Department of Agriculture (USDA) protocol (NVSL-SOP-0068.04). The RT-qPCR assays were carried out with the Qiagen One-Step RT-PCR kit ID 210212 (Qiagen, Hilden, Germany) using the Applied Biosystems 7500 Fast Real-Time PCR system.

### 2.5. RNA Extraction and Illumina Sequencing

Genome sequencing was accomplished directly from the swabs. Reverse transcription was carried out using the Superscript IV First-Strand Synthesis System (Thermo Fisher Scientific, Waltham, MA, USA).

Viral sequence enrichment was performed using a PCR primer pair that amplified all AIV segments [25]. The Nextera™ DNA Flex Library Preparation kit (Illumina, San Diego, CA, USA) was used from 100 ng of the amplicon. Libraries were purified with AMPure XP (Beckman Coulter, Indianapolis, IN, USA) and quantified with the Qubit dsDNA HS assay kit (Thermo Fisher Scientific, Waltham, MA, USA). The library’s quality and length were assessed with the Agilent high-sensitivity DNA kit (Agilent, Santa Clara, CA, USA) on a Fragment Analyzer™ System (Advanced Analytical Technologies Inc., Heidelberg, Germany). Genome sequencing was performed on the Genomic Platform at the Faculty of Science, University of the Republic (UdelaR), Uruguay, using an Illumina MiniSeq (Illumina, USA) with MiniSeqTM Mid Output Reagent Cartridge (300 cycles, paired-end reads). 

### 2.6. Genome Assembly and Annotation

The BBDuK and Minimap2 plugins in Geneious [26] were used to trim and filter the raw data and map the clean reads to an avian influenza genome from South America. Assemblies were visually inspected and optimized; annotations were transferred from reference strains and manually curated. 

### 2.7. Phylogenetic Analysis

A full-length genome dataset was compiled using South and North American strains available in the Influenza Virus Database of the National Center for Biotechnology Information. Sequences were aligned using MAFFT [27] and trimmed to the starting ATG and ending STOP codon. All groups of identical sequences in the datasets were collapsed and represented by the oldest sequence. The identification of potentially recombinant sequences was performed using the RDP4 program [28]. Seven methods were employed to explore the putative recombination events. Recombination events were positive when supported by five methods with a *p*-value adjusted to 0.05.

Maximum-likelihood trees, with approximate likelihood ratio tests for internal node support, were inferred in Geneious using FastTree [29]. Eurasian sequences were used as an outgroup. Tree visualization was performed using the R package ggtree.

## 3. Results

### 3.1. Description of the Outbreaks 

The index outbreak of avian influenza in Uruguay was detected on 14 February 2023, in Laguna Garzón, a lagoon on the border of the departments of Maldonado and Rocha with a sizeable population of migratory waterfowl. Two black-necked swans (*Cygnus melancoryphus*) found dead tested positive using RT-qPCR for the AIV matrix gene and Eurasian lineage H5. This finding led to the notification of the World Organization for Animal Health (WOAH). 

Twelve orotracheal or cloacal swabs tested positive for AIV and the H5 subtype using RT-qPCR (Table 1) and were used in this study. These included one sample from a black-necked swan (sample 014_M3) collected on 18 February 2023 from the index outbreak. A second sample (sample 078_M2) obtained from a black-necked swan found dead on Solymar Beach, Canelones department, 150 km from Laguna Garzón, was also included. The remaining samples came from five outbreaks affecting domestic chickens, ducks, and turkeys kept for egg and meat production (backyard poultry). Samples were collected from four different geographic locations up to 350 km apart (Table 1). Backyard outbreaks have affected 705 poultry, mainly chickens, with 181 deaths and the remaining being stamped out. The clinical signs included a sudden onset of up to 100% mortality, acute death, bleeding disorders, lethargy, reluctance to move, loss of appetite, diarrhea, subcutaneous hemorrhages, cyanotic, and necrotic spots, especially on combs, wattles, and legs. The deaths of black-necked swans amounted to 142 individuals in Laguna Garzón, with the most remarkable symptoms being neurological.

### 3.2. Genome Sequence Comparison 

The coding sequences of the twelve samples were successfully obtained and deposited in the GenBank database; the accession numbers are given in Table 1. The complete coding sequences of the eight viral segments have 13,069 nucleotides and include antiviral drug-resistant residues such as 31Asn (M2 protein) and 275His (NA protein). Uruguayan sequences displayed 142 nucleotide substitutions, with 89 being synonymous and 53 being non-synonymous (Table 2 and Table S1). 

The coding genome of sample 014_M3 showed some unassigned bases (~5% of the genome) in the first three segments, PB2, PB1, and PA, that could be a consequence of a lower viral titer, as reflected by a higher threshold cycle in the qPCR, or problems with sample conservation as this sample came from a dead animal (Table 1). The samples from the Department of San José (124_M1, 124_M3, and 124_M6) have identical coding genomes.

### 3.3. HA Sequence Comparison

Using the genome of the first detected AIV strain (014_M3) as a reference, Uruguayan HA sequences had twenty single nucleotide substitutions; thirteen were synonymous changes, and seven were non-synonymous (Table 2). The A99S and H289N substitutions were found in strains from the departments of San José and Tacuarembó, respectively.

Based on the BLAST search conducted in the GenBank database with the consensus sequence of Uruguayan strains, the sequences with the highest similarity (99.8 to 100%) were strains from Chile, Peru, and Argentina. 

### 3.4. HPAIV Cleavage Site 

All viruses characterized in this study have the basic amino acid motif (PLREKRRKR/G) at cleavage sites (residues 337 to 346 in HA). Most North and South American sequences have this cleavage site, except for two Venezuelan and a few North American sequences (North Carolina and Kansas) with PLRERRRKR/G and a Peruvian strain with PLREKRRGR/G.

### 3.5. Phylogenetic Analysis of North and South American Strains 

**Figure 1:**
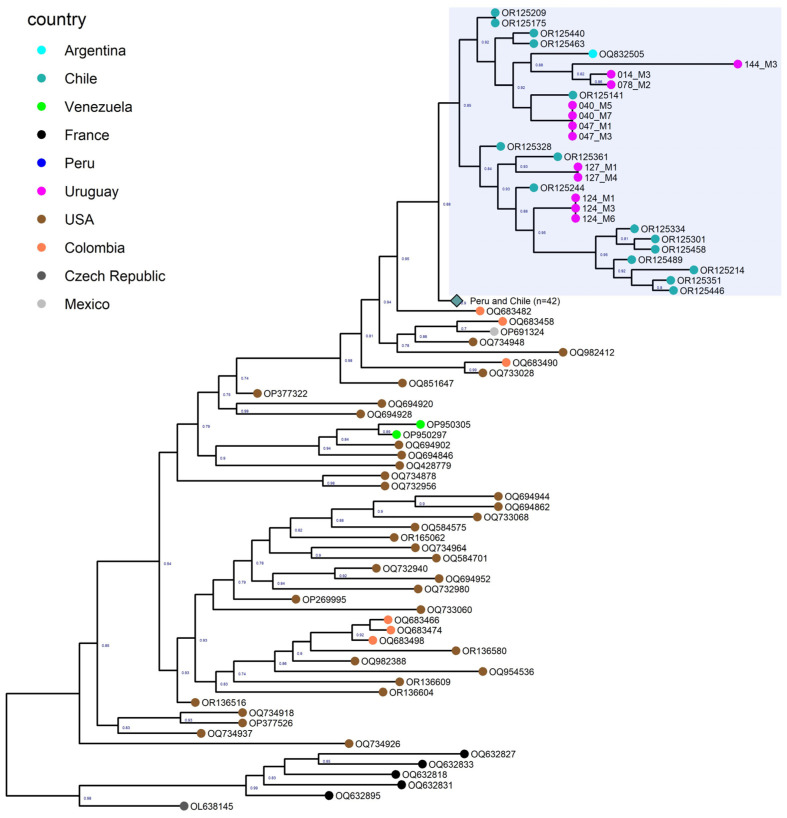
Maximum-likelihood tree based on the hemagglutinin coding regions of different avian influenza strains. Uruguayan strains are highlighted in sky blue.

The Uruguayan strains were closely associated with strains from Argentina and Chile (Figure 1). Peruvian and other Chilean strains formed a sister clade basal to this group. A Colombian strain is basal to this group. Other Colombian strains were situated at tree positions on the phylogenetic tree related to North American (Mexico and United States) strains. Venezuela strains fell in a different position of the phylogenetic tree and are closely linked with strains from the United States. 

### 3.6. Phylogenetic Analysis of the Uruguayan Strains-Containing Clade 

**Figure 2:**
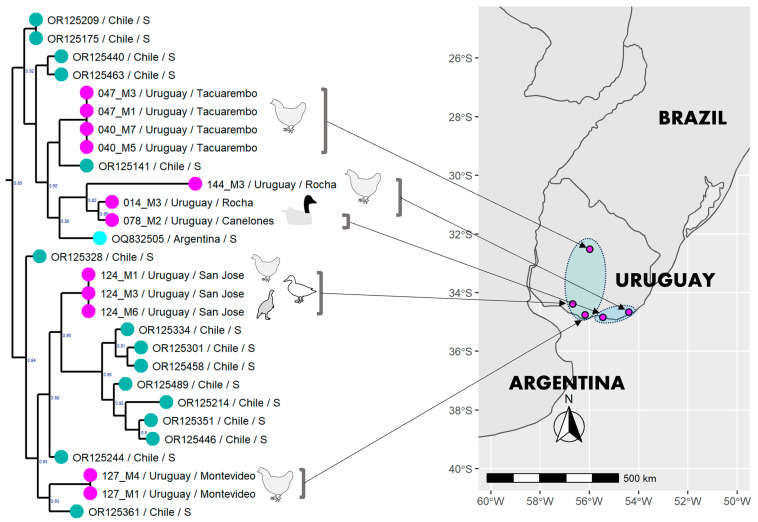
Origin and phylogenetic relationship of the AIV strains. The Uruguayan clade from the maximum-likelihood HA tree has been linked to a country map to show where the samples originated.

The Uruguayan strains belong to a clade comprising strains from the southern regions of Argentina and Chile, interspersed with the Uruguayan ones (Figure 1 and Figure 2). The strains from the south and east regions of Uruguay (Canelones and Rocha) are associated with the only strain from Argentina (only the HA and NA segments were available). The strains from the center and south (Tacuarembó, San José, and Montevideo) fell into different subclades associated with strains from the south of Chile. The Uruguayan strains are not associated based on the host species (swan, chicken, duck, and turkey) (Figure 2). 

### 3.7. Other Segments Comparison

**Figure 3:**
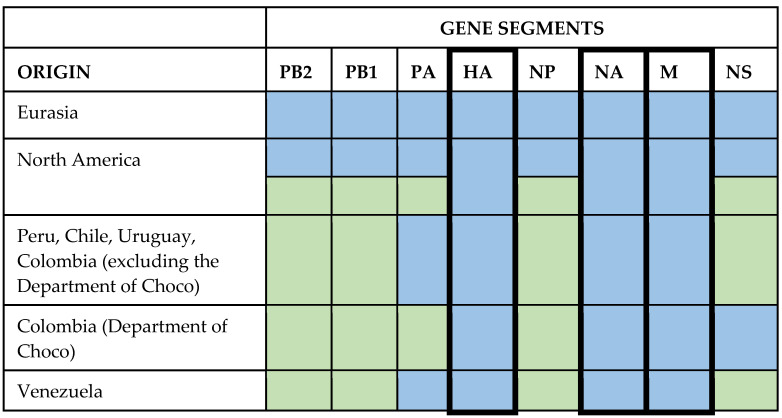
Diagram depicting the origin of segments in AIV 2.3.4.4b strains. The accession numbers of all sequences used for this analysis are included in Table S2. The color green represents Eurasian origin, while the color sky blue represents American origin.

A phylogenetic analysis with the seven remaining segments showed similar relationships for the Uruguayan strains associated with Chilean and Peruvian strains (Figure 3 and Figure S1). 

Some segments showed reassortment events, as evidenced by monophyletic clades with large branch distances and significant nucleotide divergence (Supplementary Figure S1). The reassortment occurred between strains of Eurasian origin that migrated to America and were reshuffled with endemic strains to generate segments of North American origin. 

All viruses from America retained the parental European-origin HA, NA, and M segments (i.e., they are not reassortants for these segments). However, South American strains had specific combinations of the polymerase, NP, and NS segments of European or North American origin (Figure 3). South American strains had PB1 and PB2 segments of North American origin. The PA segment was of Eurasian origin, except for Colombian strains from the Department of Choco, which had North American origin. These Colombian strains were associated with strains from the United States of North American origin. All South American strains possessed the NP and NS segments of North American origin, except for Colombian strains from the Department of Choco that retained the NS segment of Eurasian origin. 

## 4. Discussion

### 4.1. Detection and Genomic Characterization

This study comprehensively analyzes the first avian influenza virus outbreaks in Uruguay, focusing on its genetic characterization and relationship with high-pathogenicity avian influenza in South and North America. The outbreaks occurred consecutively in Uruguay’s central, southern, and eastern regions and were confirmed by qPCR in twelve samples from different species of wild birds and backyard poultry (Table 1). 

The phylogenetic analysis demonstrates that the twelve fully sequenced viruses belong to the H5 clade 2.3.4.4b and cluster with viruses from wild bird species in America (Figure 1). The HA phylogeny indicates that Uruguayan viruses have a common ancestor with those of Argentina, Chile, and Peru. This association remains apparent in the phylogenetic analysis performed for all the segments. Basal to this monophyletic group are the South American (Colombia) and North American strains (the United States and Mexico) in a basal position. The Colombian strains occupy different positions in the phylogenetic tree, with the strains from the Department of Choco (index case in Colombia) located most distant. The Venezuelan strains are associated with the United States strains in a different position of the phylogenetic tree. The different positions in the HA phylogeny of Colombian and Venezuelan strains and their association with strains from various North American regions support that these countries experienced multiple AIV introductions [30]. 

### 4.2. Local Divergence

The viruses collected from nearby areas in Uruguay during the same time frame show a close genetic relationship using the HA sequence (Table 1, Figure 2). Additionally, samples from various hosts, such as turkey, duck, and chicken, from the same outbreak (Department of San José) have identical sequences and amino acid markers in the coding genome. Based on the analysis within a limited time frame, the virus may diverge based on spatiotemporal factors rather than being exclusive to a particular host. All the backyard poultry resided near ponds or small lagoons, where they often interacted with waterfowl, gulls, and shorebirds. This agrees with the scenario where HPAIV is introduced into poultry and backyard populations through the activity of wild aquatic birds. The spread of the virus in poultry populations can occur through indirect contact; waterfowl can transmit the virus to an area where it may spread to poultry through other contaminated animal feces. 

### 4.3. Pathogenicity

All viruses analyzed here share the multibasic PLREKRRKR/G cleavage site, indicative of a highly pathogenic phenotype in chickens. The clinical severity in other hosts varies with bird species and the genetic characteristics of the strains. The Uruguayan strains have been shown to cause extremely rapid deaths in poultry and wild black-necked swans (*Cygnus melancoryphus*). This species has been previously reported to be susceptible to influenza [31]. According to recent studies, swans possess certain genome traits that make them more vulnerable to influenza [32]. High pathogenicity has been observed in other South American wild birds. In Perú, the virus killed more than 50,000 wild birds by the end of 2022, particularly Peruvian pelicans (*Pelecanus thagus*) and Peruvian boobies (*Sula variegata*). The large biomass of infected wild birds may have led to a spillover event affecting predators and scavengers, including marine mammals cohabiting with them, as reported in other parts of the world [33,34,35]. 

All viruses had the Ser31Asn change in the M2 protein and the 275His change in the NA protein, which confer resistance to amantadine [36] and oseltamivir [37].

### 4.4. Reassortments in American Strains

There are different viral genotypes among the South American strains. The HA, NA, and M segments had Eurasian origin and did not undergo reassortment with other viruses. The remaining segments underwent reassortment with viruses of North American origin. The North American gene segments are monophyletic, suggesting that a single or minimal number of reassortment events have occurred, with the resulting viruses spreading geographically [38]. 

South American strains have the PB2, PB1, NP, and NS segments of North American origin, except for the NS segment of Colombian strains from the Department of Choco (index cases in Colombia), which retained its Eurasian origin. The PA segment is of Eurasian origin, except for the Choco strains, which are of North American origin. The observed reassortment patterns in Colombian strains provide additional evidence for the hypothesis that the avian influenza virus in the country may have originated from different sources (Figure 3) [30].

Based on these findings, North America has two genotypes (the ancestral and the resorted one). In contrast, South America has three (Venezuela, the Choco region of Colombia, and the countries of Peru, Chile, Colombia (excluding Choco), and Uruguay).

### 4.5. Spreading in South America through the Pacific Flyway

The American H5N1 emerged from 2.3.4.4b H5N8 along the Adriatic Flyway during mid-2020 and repeatedly acquired internal genes through reassortment with LPAI [39]. These strains first arrived in Canada in 2021 and rapidly spread to the United States via wild birds in the Atlantic Flyway. Compared to their ancestors, this lineage underwent two amino acid changes (L120M and I526V) in mid-2021 [39]. These HA substitutions occurred in Uruguayan viruses; L120M is conserved in America, but I526V is variable. 

The first cases in South America were reported in October 2022 in Acandí, a municipality in the Colombian department of Chocó, on the Caribbean Sea of the Atlantic Ocean. This outbreak involved captive domestic and wild birds from a farm less than 10 km from Panama. This initial case was followed by outbreaks in Peru, Venezuela, and Ecuador (November 2022), Chile (December 2022), Bolivia (January 2023), Argentina and Uruguay (February 2023), and Paraguay and Brazil (May 2022) [40]. 

Based on the spread pattern, the initial detection in Colombia may have occurred within the Atlantic Flyway, but the virus eventually spread throughout South America via the Pacific Flyway [40]. 

The first case of HPAI H5N1 in Uruguay was reported in Laguna Garzón, a coastal lagoon that forms part of a vast system of marine waters on the Atlantic coast of the Southern Cone. Uruguay, located in the southeastern region of South America and bordered by Argentina and Brazil along the Atlantic Ocean, may have been considered a potential route for the Atlantic Flyway. Nevertheless, the similarity of the Uruguayan strains with Argentine and Chilean viruses indicates that the virus arrived following the initial Pacific Flyway and later spread to Uruguay. Uruguay is not reached by birds migrating on the Pacific Flyway, and the virus needs to be transported across the Andes to enter Uruguay. This Andean trespassing could have been mediated by several species of bird from the high-altitude lagoons, including the Andean geese (*Chloephaga melanoptera*) and the austral flamingo, *Phoenicopterus chilensis*, a visitor of Uruguay in the coastal lagoons. The virus might have entered Uruguay via the fluvial, lagoon, and wetland systems that connect to the Pilcomayo, Bermejo, and Salado del Norte rivers [41].

Based on their close phylogenetic relationship (Figure 2), it is likely that the cases of swan infection in the Rocha department in February 2023 and in the Canelones department one month later were of Argentine origin. Both Uruguayan sequences were obtained from swans corresponded to *Cygnus melancoryphus*, a nomadic species that moves opportunistically. This species does not undertake seasonal long-distance migrations like migrant swans in the northern hemisphere but redistributes in response to local conditions [42]. These movements may result from environmental threats, including the rapid loss of wetland habitat associated with the recent drought that has affected Uruguay and some Argentine regions [43]. These swans or co-inhabiting aquatic birds could have posteriorly (May 2023) transmitted the virus to backyard chickens in the Department of Rocha (Figure 2). 

On the contrary, strains from central (Department of Tacuarembó) and southern Uruguay (Departments of Montevideo and San José) might have a different origin from Chilean strains (Figure 2). Unfortunately, there are no other sequences from other regions in Argentina to support this hypothesis.

### 4.6. Avian Influenza Poses a Significant Risk to the Poultry Sector in South America

Over the last few decades, the South American poultry industry has grown substantially, and many countries in the region, such as Argentina, Brazil, Chile, Colombia, and Peru, have established themselves as major players in the global market for poultry. The industry primarily focuses on producing chicken and eggs, with Brazil being the world’s largest producer and exporter of chicken meat. The growth of this sector is attributed to various factors such as increased demand for poultry products, favorable weather conditions, resource availability, government policies, and good sanitary status. However, avian influenza has become a significant threat with devastating consequences for the poultry industry. 

The worldwide spread of AIV exemplified the synergy between wild and domestic interphases and wild bird movement, including migrations, post-migration movements, or even daily movements [44]. Since domestic poultry can share the same habitat, water, and food as wild waterfowl, their presence and concentration make them critical intermediate hosts between wild birds and poultry. 

The overlap of the migratory movements of wild waterbirds along the Pacific coast of South America with densely populated poultry areas may increase the risk of viral incursion into poultry farms, emphasizing the need to implement effective biosecurity measures and strategically plan the spatial layout of the poultry industry. It is necessary to mitigate endemic disease in poultry to avoid these production systems potentially acting as future sources for emerging variants. 

In the present scenario, isolating backyard and commercial poultry from wildlife is one of the best options to fight against the virus. Rearing backyard poultry in isolation demands a cultural shift in traditional animal-keeping practices for those living in rural areas. It is crucial to prioritize a One Health approach that focuses on human and animal welfare and the sustainability of ecosystems to control avian influenza effectively. 

## Figures and Tables

**Table 1:** Epidemiological data of the avian influenza virus samples from Uruguay. Accession numbers are indicated.

Date	Sample	Origin	Species	RT-qPCR AIV Matrix (Cq)	Accession
18 February 2023	014_M3	Rocha (Laguna de Garzón)	Wild, black-necked swan	26.5–27.8	OR381584-OR381591
3 March 2023	040_M5	Tacuarembó	Backyard chicken	25.2	OR381592-OR381599
3 March 2023	040_M7	Tacuarembó	Backyard chicken	22.6	OR381600-OR381607
6 March 2023	047_M1	Tacuarembó	Backyard chicken	18.2	OR381608-OR381615
6 March 2023	047_M3	Tacuarembó	Backyard chicken	18.9	OR381616-OR381623
15 March 2023	078_M2	Canelones (Solymar)	Wild, black-necked swan	25	OR381672-OR381679
16 April 2023	124_M1	San José	Backyard chicken	20.6	OR381624-OR381631
16 April 2023	124_M3	San José	Backyard duck	19.5	OR381656-OR381663
16 April 2023	124_M6	San José	Backyard turkey	29.1	OR381664-OR381671
17 April 2023	127_M1	Montevideo	Backyard chicken	22.1–27.5	OR381632-OR381639
17 April 2023	127_M4	Montevideo	Backyard chicken	22.1–27.5	OR381640-OR381647
3 May 2023	144_M3	Rocha	Backyard chicken	15.8	OR381648-OR381655

**Table 2:** Single nucleotide polymorphisms (SNP) in the genome segments of the avian influenza virus from Uruguay. The number of synonymous and non-synonymous changes and the coding sequence length of each segment are provided. Only a PB2 sequence from Chile has a 3-nt insertion; one Peruvian NS sequence has a deletion.

	PB2	PB1	PA	HA	NP	NA	M	NS1/NEP	Total
Nt (CDS)	2213	2274	2151	1704 (18 aa signal peptide)	1497	1410	982	838 (NS) 693/366	13.069
SNP	26	27	21	20	14	14	7	13	142
Syn	17	21	11	13	10	9	2	6	89
Non-syn	9	6	10	7	4	5	5	7	53
Sequences with nnns (length)	014_M3 (294)	014_M3 (427 nt)	014_M3 (22 nt)						

## Data Availability

All sequence data generated in this study were submitted to the GenBank database (Accession numbers are listed in Table 1).

## Supplementary Materials

The following supporting information can be downloaded at https://www.mdpi.com/article/10.3390/v15091906/s1, Table S1: Nucleotide variants observed in the Uruguayan sequences. The nucleotide change (Change), the position in the coding sequence (CDS) and in the codon (Codon number), and the number of sequences (n°) with the change are indicated. The amino acid (AA) change and its effect on the protein (Protein effect) are also shown for each single nucleotide polymorphism (SNP); Table S2: Sequences used for reassortment analysis showed in Figure 3. Accession numbers (Accession) for the eight avian influenza segments (Segment), country of origin (Country). the strain name (Strains) and the host species (Host) are indicated. Figure S1: phylogenies.

## References

[1] Lefkowitz E.J., Dempsey D.M., Hendrickson R.C., Orton R.J., Siddell S.G., Smith D.B. (2018). Virus taxonomy: The database of the International Committee on Taxonomy of Viruses (ICTV). Nucleic Acids Res..

[2] Spackman E. (2008). A Brief Introduction to the Avian Influenza Virus. Avian Influenza Virus.

[3] Mostafa A., Abdelwhab E.M., Mettenleiter T.C., Pleschka S. (2018). Zoonotic Potential of Influenza A Viruses: A Comprehensive Overview. Viruses.

[4] Duffy S. (2008). Why are RNA virus mutation rates so damn high?. PLoS Biol..

[5] Kuiken T. (2013). Is low pathogenic avian influenza virus virulent for wild waterbirds?. Proc. R. Soc. B Biol. Sci..

[6] Olsen B., Munster V.J., Wallensten A., Waldenström J., Osterhaus A.D., Fouchier R.A. (2006). Global Patterns of Influenza A Virus in Wild Birds. Science.

[7] Webster R.G., Yakhno M., Hinshaw V.S., Bean W.J., Murti K.C. (1978). Intestinal influenza: Replication and characterization of influenza viruses in ducks. Virology.

[8] Wan X.F. (2012). Lessons from Emergence of A/Goose/Guangdong/1996-Like H5N1 Highly Pathogenic Avian Influenza Viruses and Recent Influenza Surveillance Efforts in Southern China. Zoonoses Public Health.

[9] Xu X., Subbarao K., Cox N.J., Guo Y. (1999). Genetic Characterization of the Pathogenic Influenza A/Goose/Guangdong/1/96 (H5N1) Virus: Similarity of Its Hemagglutinin Gene to Those of H5N1 Viruses from the 1997 Outbreaks in Hong Kong. Virology.

[10] Hill N.J., Hussein I.T., Davis K.R., Ma E.J., Spivey T.J., Ramey A.M., Puryear W.B., Das S.R., Halpin R.A., Lin X. (2017). Reassortment of influenza a viruses in wild birds in alaska before H5 clade 2.3.4.4 outbreaks. Emerg. Infect. Dis..

[11] Shao W., Li X., Goraya M.U., Wang S., Chen J.-L. (2017). Evolution of Influenza A Virus by Mutation and Re-Assortment. Int. J. Mol. Sci..

[12] Dhingra M.S., Artois J., Robinson T.P., Linard C., Chaiban C., Xenarios I., Engler R., Liechti R., Kuznetsov D., Xiao X. (2016). Global mapping of highly pathogenic avian influenza H5N1 and H5Nx clade 2.3.4.4 viruses with spatial cross-validation. eLife.

[13] Antigua K.J.C., Choi W.-S., Baek Y.H., Song M.-S. (2019). The Emergence and Decennary Distribution of Clade 2.3.4.4 HPAI H5Nx. Microorganisms.

[14] Lewis N.S., Banyard A.C., Whittard E., Karibayev T., Al Kafagi T., Chvala I., Byrne A., Meruyert S., King J., Harder T. (2021). Emergence and spread of novel H5N8, H5N5 and H5N1 clade 2.3.4.4 highly pathogenic avian influenza in 2020. Emerg. Microbes Infect..

[15] Gu W., Shi J., Cui P., Yan C., Zhang Y., Wang C., Zhang Y., Xing X., Zeng X., Liu L. (2022). Novel H5N6 reassortants bearing the clade 2.3.4.4b HA gene of H5N8 virus have been detected in poultry and caused multiple human infections in China. Emerg. Microbes Infect..

[16] Bevins S.N., Shriner S.A., Cumbee J.C., Dilione K.E., Douglass K.E., Ellis J.W., Killian M.L., Torchetti M.K., Lenoch J.B. (2022). Intercontinental Movement of Highly Pathogenic Avian Influenza A(H5N1) Clade 2.3.4.4 Virus to the United States, 2021. Emerg. Infect. Dis..

[17] Adlhoch C., Fusaro A., Gonzales J.L., Kuiken T., Marangon S., Niqueux É., Staubach C., Terregino C., Aznar I., Guajardo I.M. (2022). Avian influenza overview December 2021–March 2022. EFSA J..

[18] Caliendo V., Lewis N.S., Pohlmann A., Baillie S.R., Banyard A.C., Beer M., Brown I.H., Fouchier R.A.M., Hansen R.D.E., Lameris T.K. (2022). Transatlantic spread of highly pathogenic avian influenza H5N1 by wild birds from Europe to North America in 2021. Sci. Rep..

[19] Bruno A., Alfaro-Núñez A., de Mora D., Armas R., Olmedo M., Garcés J., Muñoz-López G., Garcia-Bereguiain M.A. (2023). First case of human infection with highly pathogenic H5 avian influenza a virus in South America: A new zoonotic pandemic threat for 2023?. J. Travel Med..

[20] Puryear W., Sawatzki K., Hill N., Foss A., Stone J.J., Doughty L., Walk D., Gilbert K., Murray M., Cox E. (2023). Highly Pathogenic Avian Influenza A(H5N1) Virus Outbreak in New England Seals, United States. Emerg. Infect. Dis..

[21] Günther A., Krone O., Svansson V., Pohlmann A., King J., Hallgrimsson G.T., Skarphéðinsson K.H., Sigurðardóttir H., Jónsson S.R., Beer M. (2022). Iceland as Stepping Stone for Spread of Highly Pathogenic Avian Influenza Virus between Europe and North America. Emerg. Infect. Dis..

[22] Ariyama N., Pardo-Roa C., Munoz G., Aguayo C., Avila C., Mathieu C., Brito B., Medina R., Johow M., Neira-Ramirez V. (2023). Emergence and rapid dissemination of highly pathogenic avian influenza virus H5N1 clade 2.3.4.4b in wild birds, Chile. bioRxiv.

[23] Adlhoch C., Fusaro A., Gonzales J.L., Kuiken T., Marangon S., Mirinaviciute G., Niqueux É., Stahl K., Staubach C., Terregino C. (2023). Avian influenza overview December 2022–March 2023. EFSA J..

[24] Gamarra-Toledo V., Plaza P.I., Gutiérrez R., Luyo P., Hernani L., Angulo F., Lambertucci S.A. (2023). Avian flu threatens Neotropical birds. Science.

[25] Zhou B., Donnelly M.E., Scholes D.T., George K.S., Hatta M., Kawaoka Y., Wentworth D.E. (2009). Single-Reaction Genomic Amplification Accelerates Sequencing and Vaccine Production for Classical and Swine Origin Human Influenza A Viruses. J. Virol..

[26] Kearse M., Moir R., Wilson A., Stones-Havas S., Cheung M., Sturrock S., Buxton S., Cooper A., Markowitz S., Duran C. (2012). Geneious Basic: An integrated and extendable desktop software platform for the organization and analysis of sequence data. Bioinformatics.

[27] Katoh K., Standley D.M. (2013). MAFFT Multiple Sequence Alignment Software Version 7: Improvements in Performance and Usability. Mol. Biol. Evol..

[28] Martin D.P., Murrell B., Golden M., Khoosal A., Muhire B. (2015). RDP4: Detection and analysis of recombination patterns in virus genomes. Virus Evol..

[29] Price M.N., Dehal P.S., Arkin A.P. (2009). FastTree: Computing Large Minimum Evolution Trees with Profiles instead of a Distance Matrix. Mol. Biol. Evol..

[30] Ruiz-Saenz J., Martinez-Gutierrez M., Pujol F.H. (2023). Multiple introductions of highly pathogenic avian influenza H5N1 clade 2.3.4.4b into South America. Travel Med. Infect. Dis..

[31] Ellis T.M., Bousfield R.B., Bissett L.A., Dyrting K.C., Luk G.S.M., Tsim S.T., Sturm-Ramirez K., Webster R.G., Guan Y., Peiris J.S.M. (2004). Investigation of outbreaks of highly pathogenic H5N1 avian influenza in waterfowl and wild birds in Hong Kong in late 2002. Avian Pathol..

[32] Karawita A.C., Cheng Y., Chew K.Y., Challagulla A., Kraus R., Mueller R.C., Tong M.Z.W., Hulme K.D., Bielefeldt-Ohmann H., Steele L.E. (2023). The swan genome and transcriptome, it is not all black and white. Genome Biol..

[33] Castro-Sanguinetti G., Gonzalez-Veliz R., Callupe-Leyva A., Apaza-Chiara A., Jara J., Silva W., Icochea E., More-Bayona J. (2023). Circulation of highly pathogenic avian influenza virus H5N1 clade 2.3.4.4b in highly diverse wild bird species from Peru. Res. Sq..

[34] Leguia M., Garcia-Glaessner A., Muñoz-Saavedra B., Juarez D., Barrera P., Calvo-Mac C., Jara J., Silva W., Ploog K., Amaro L. (2023). Highly pathogenic avian influenza A (H5N1) in marine mammals and seabirds in Peru. bioRxiv.

[35] Jimenez-Bluhm P., Siegers J.Y., Tan S., Sharp B., Freiden P., Johow M., Orozco K., Ruiz S., Baumberger C., Galdames P. (2023). Detection and Phylogenetic Analysis of Highly Pathogenic A/H5N1 Avian Influenza Clade 2.3.4.4b Virus in Chile, 2022. Emerg. Microbes Infect..

[36] Scholtissek C., Quack G., Klenk H.D., Webster R.G. (1998). How to overcome resistance of influenza A viruses against adamantane derivatives. Antivir. Res..

[37] Treanor J.J., Hayden F.G., Vrooman P.S., Barbarash R., Bettis R., Riff D., Singh S., Kinnersley N., Ward P., Mills R.G. (2000). Efficacy and Safety of the Oral Neuraminidase Inhibitor Oseltamivir in Treating Acute Influenza. JAMA.

[38] Kandeil A., Patton C., Jones J.C., Jeevan T., Harrington W.N., Trifkovic S., Seiler J.P., Fabrizio T., Woodard K., Turner J.C. (2023). Rapid evolution of A(H5N1) influenza viruses after intercontinental spread to North America. Nat. Commun..

[39] Xie R., Edwards K.M., Wille M., Wei X., Wong S.S., Zanin M., El-Shesheny R., Ducatez M., Poon L.L., Kayali G. (2022). The episodic resurgence of highly pathogenic avian influenza H5 virus. bioRxiv.

[40] PAHO/WHO (2023). Epidemiological Update Outbreaks of Avian Influenza Caused by Influenza A(H5N1) in the Region of the Americas.

[41] Capllonch P. (2018). Un panorama de las migraciones de aves en Argentina. El Hornero.

[42] Schlatter R.P., Navarro R.A., Corti P. (2002). Effects of El Nino Southern Oscillation on Numbers of Black-Necked Swans at Rio Cruces Sanctuary, Chile. Waterbirds.

[43] Rees E.C., Clausen P., Coleman J.T. (2019). Conservation status of the world’s swan populations, *Cygnus* sp. and *Coscoroba* sp.: A review of current trends and gaps in knowledge. Wildfowl.

[44] Teitelbaum C.S., Casazza M.L., McDuie F., De La Cruz S.E.W., Overton C.T., Hall L.A., Matchett E.L., Ackerman J.T., Sullivan J.D., Ramey A.M. (2023). Waterfowl recently infected with low pathogenic avian influenza exhibit reduced local movement and delayed migration. Ecosphere.

