# Supplementary material for: Spreading of the High-Pathogenicity Avian Influenza (H5N1) Virus of Clade 2.3.4.4b into Uruguay
Source: Viruses. 2023 Sep 11;15(9):1906. doi: 10.3390/v15091906 (PMC10536905; doi:10.3390/v15091906)
Supplement: Supplementary file 1 [file viruses-15-01906-s001.zip › Table S1.pdf]

Table S1. Nucleotide variants observed in the Uruguayan sequences. The nucleotide change (Change), the position in the coding sequence (CDS) and in the codon (Codon number), and the number of sequences (nº) with the change are indicated. The amino acid (AA) change and its effect on the protein (Protein effect) are also shown for each single nucleotide polymorphism (SNP).

gene	Change	CDS position	Codon number	Codon change	AA change	Polymorphism.Typ	Protein effect	nº
HA	T -> C	32	11	GTT -> GCT	V -> A	SNP (transition)	Substitution	1
HA	G -> A	191	64	AGG -> AAG	R -> K	SNP (transition)	Substitution	11
HA	A -> C	205	69	AAG -> CAG	K -> Q	SNP (transversion)	Substitution	1
HA	T -> C	216	72	AGT -> AGC		SNP (transition)	None	1
HA	G -> A	263	88	AGA -> AAA	R -> K	SNP (transition)	Substitution	1
HA	G -> T	295	99	GCT -> TCT	A -> S	SNP (transversion)	Substitution	3
HA	C -> T	357	119	CAC -> CAT		SNP (transition)	None	1
HA	G -> T	468	156	GCG -> GCT		SNP (transversion)	None	5
HA	T -> C	585	195	CAT -> CAC		SNP (transition)	None	2
HA	T -> C	786	262	AAT -> AAC		SNP (transition)	None	3
HA	C -> A	865	289	CAC -> AAC	H -> N	SNP (transversion)	Substitution	4
HA	G -> A	1143	381	GCG -> GCA		SNP (transition)	None	4
HA	T -> C	1185	395	AAT -> AAC		SNP (transition)	None	1
HA	C -> T	1194	398	AAC -> AAT		SNP (transition)	None	1
HA	A -> G	1201	401	ATT -> GTT	I -> V	SNP (transition)	Substitution	1
HA	C -> T	1218	406	ACC -> ACT		SNP (transition)	None	9
HA	C -> T	1251	417	AAC -> AAT		SNP (transition)	None	2
HA	T -> C	1317	439	TAT -> TAC		SNP (transition)	None	2
HA	C -> T	1440	480	AAC -> AAT		SNP (transition)	None	1
HA	A -> G	1503	501	ACA -> ACG		SNP (transition)	None	9
M2	A -> G	103	35	ATC -> GTC	I -> V	SNP (transition)	Substitution	2
M2	G -> A	151	51	GTT -> ATT	V -> I	SNP (transition)	Substitution	5
M1	G -> A	343	115	GTT -> ATT	V -> I	SNP (transition)	Substitution	4
M1	C -> T	435	145	GGC -> GGT		SNP (transition)	None	2
M1	C -> G	500	167	ACC -> AGC	T -> S	SNP (transversion)	Substitution	1
M1	G -> A	544	182	GCC -> ACC	A -> T	SNP (transition)	Substitution	1
M1	C -> A	681	227	GCC -> GCA		SNP (transversion)	None	4
NA	G -> A	55	19	GTA -> ATA	V -> I	SNP (transition)	Substitution	11
NA	C -> T	90	30	ATC -> ATT		SNP (transition)	None	5
NA	T -> C	130	44	TAC -> CAC	Y -> H	SNP (transition)	Substitution	4
NA	C -> T	180	60	ACC -> ACT		SNP (transition)	None	1
NA	A -> T	202	68	AAC -> TAC	N -> Y	SNP (transversion)	Substitution	4
NA	C -> T	415	139	CTG -> TTG		SNP (transition)	None	2
NA	G -> A	474	158	TTG -> TTA		SNP (transition)	None	3
NA	A -> C	576	192	ACA -> ACC		SNP (transversion)	None	2
NA	C -> T	600	200	AAC -> AAT		SNP (transition)	None	9
NA	G -> A	850	284	GAT -> AAT	D -> N	SNP (transition)	Substitution	1
NA	C -> T	918	306	TTC -> TTT		SNP (transition)	None	11

NA	C -> A	1019	340	TCT -> TAT	S -> Y	SNP (transversion)	Substitution	3
NA	T -> C	1191	397	ACT -> ACC		SNP (transition)	None	1
NA	C -> T	1353	451	GAC -> GAT		SNP (transition)	None	1
NP	T -> C	9	3	TCT -> TCC		SNP (transition)	None	2
NP	A -> G	72	24	GAA -> GAG		SNP (transition)	None	1
NP	T -> A	99	33	GTT -> GTA		SNP (transversion)	None	1
NP	G -> A	219	73	GAG -> GAA		SNP (transition)	None	2
NP	C -> T	327	109	ATC -> ATT		SNP (transition)	None	3
NP	C -> A	387	129	GCC -> GCA		SNP (transversion)	None	11
NP	G -> A	600	200	GGG -> GGA		SNP (transition)	None	1
NP	G -> A	738	246	CGG -> CGA		SNP (transition)	None	3
NP	T -> G	967	323	TCT -> GCT	S -> A	SNP (transversion)	Substitution	9
NP	A -> G	1109	370	AAC -> AGC	N -> S	SNP (transition)	Substitution	4
NP	A -> G	1184	395	AAC -> AGC	N -> S	SNP (transition)	Substitution	4
NP	C -> T	1191	397	AAC -> AAT		SNP (transition)	None	3
NP	T -> A	1259	420	TTC -> TAC	F -> Y	SNP (transversion)	Substitution	1
NP	A -> G	1293	431	GGA -> GGG		SNP (transition)	None	3
NS1	T -> C	65	22	TTT -> TCT	F -> S	SNP (transition)	Substitution	1
NS1	T -> C	99	33	CTT -> CTC		SNP (transition)	None	2
NS1	C -> A	111	37	CGC -> CGA		SNP (transversion)	None	4
NS1	A -> G	129	43	CTA -> CTG		SNP (transition)	None	2
NEP	C -> A	133	45	CTT -> ATT	L -> I	SNP (transversion)	Substitution	1
NEP	A -> G	159	53	GGA -> GGG		SNP (transition)	None	4
NS1	C -> T	222	74	GAC -> GAT		SNP (transition)	None	1
NS1	C -> A	254	85	CCT -> CAT	P -> H	SNP (transversion)	Substitution	4
NS1	C -> T	262	88	CGC -> TGC	R -> C	SNP (transition)	Substitution	2
NEP	A -> C	265	89	ATC -> CTC	I -> L	SNP (transversion)	Substitution	4
NEP	T -> C	279	93	AGT -> AGC		SNP (transition)	None	9
NS1	A -> G	330	110	AAA -> AAG		SNP (transition)	None	3
NS1	A -> G	367	123	ATT -> GTT	I -> V	SNP (transition)	Substitution	1
NS1	C -> A	605	202	GCT -> GAT	A -> D	SNP (transversion)	Substitution	1
NS1	A -> G	631	211	AGA -> GGA	R -> G	SNP (transition)	Substitution	4
PA	A -> G	21	7	CAA -> CAG		SNP (transition)	None	2
PA	A -> C	168	56	GAA -> GAC	E -> D	SNP (transversion)	Substitution	1
PA	G -> A	364	122	GTA -> ATA	V -> I	SNP (transition)	Substitution	3
PA	C -> T	438	146	CAC -> CAT		SNP (transition)	None	2
PA	A -> G	510	170	AGA -> AGG		SNP (transition)	None	1
PA	C -> T	645	215	GCC -> GCT		SNP (transition)	None	5
PA	C -> T	648	216	GAC -> GAT		SNP (transition)	None	1

PA	C -> T	1077	359	AAC -> AAT		SNP (transition)	None	2
PA	A -> G	1213	405	AGC -> GGC	S -> G	SNP (transition)	Substitution	1
PA	A -> T	1254	418	ACA -> ACT		SNP (transversion)	None	1
PA	C -> T	1273	425	CTT -> TTT	L -> F	SNP (transition)	Substitution	1
PA	C -> T	1364	455	GCC -> GTC	A -> V	SNP (transition)	Substitution	2
PA	C -> G	1437	479	GAC -> GAG	D -> E	SNP (transversion)	Substitution	5
PA	C -> G	1658	553	GCA -> GGA	A -> G	SNP (transversion)	Substitution	1
PA	G -> A	1676	559	AGG -> AAG	R -> K	SNP (transition)	Substitution	1
PA	C -> G	1734	578	GGC -> GGG		SNP (transversion)	None	3
PA	G -> A	1749	583	CGG -> CGA		SNP (transition)	None	8
PA	C -> T	1753	585	CTT -> TTT	L -> F	SNP (transition)	Substitution	1
PA	A -> G	1808	603	AAA -> AGA	K -> R	SNP (transition)	Substitution	1
PA	T -> C	1956	652	TCT -> TCC		SNP (transition)	None	3
PA	T -> C	1993	665	TTG -> CTG		SNP (transition)	None	1
PB1	T -> C	6	2	TAT -> TAC		SNP (transition)	None	1
PB1	A -> G	85	29	AAA -> GAA	K -> E	SNP (transition)	Substitution	9
PB1	A -> G	93	31	GGA -> GGG		SNP (transition)	None	9
PB1	A -> G	100	34	ACA -> GCA	T -> A	SNP (transition)	Substitution	4
PB1	G -> T	297	99	AAG -> AAT	K -> N	SNP (transversion)	Substitution	1
PB1	C -> T	336	112	AAC -> AAT		SNP (transition)	None	5
PB1	C -> A	582	194	AGC -> AGA	S -> R	SNP (transversion)	Substitution	2
PB1	A -> T	837	279	ACA -> ACT		SNP (transversion)	None	1
PB1	A -> G	976	326	ATG -> GTG	M -> V	SNP (transition)	Substitution	3
PB1	G -> A	1014	338	AAG -> AAA		SNP (transition)	None	2
PB1	A -> G	1092	364	AGA -> AGG		SNP (transition)	None	2
PB1	A -> G	1130	377	GAT -> GGT	D -> G	SNP (transition)	Substitution	1
PB1	A -> G	1224	408	AGA -> AGG		SNP (transition)	None	9
PB1	A -> G	1308	436	GGA -> GGG		SNP (transition)	None	4
PB1	C -> A	1353	451	GTC -> GTA		SNP (transversion)	None	1
PB1	C -> T	1428	476	CGC -> CGT		SNP (transition)	None	1
PB1	A -> G	1653	551	TCA -> TCG		SNP (transition)	None	2
PB1	A -> G	1698	566	GGA -> GGG		SNP (transition)	None	9
PB1	C -> T	1785	595	GAC -> GAT		SNP (transition)	None	4
PB1	T -> C	1794	598	TAT -> TAC		SNP (transition)	None	2
PB1	A -> G	1803	601	AGA -> AGG		SNP (transition)	None	9
PB1	G -> A	1824	608	CCG -> CCA		SNP (transition)	None	1
PB1	C -> T	1989	663	GAC -> GAT		SNP (transition)	None	1
PB1	T -> C	2157	719	GCT -> GCC		SNP (transition)	None	3
PB2	G -> A	138	46	GAG -> GAA		SNP (transition)	None	1

PB2	T -> C	180	60	GAT -> GAC		SNP (transition)	None	5
PB2	G -> A	243	81	CCG -> CCA		SNP (transition)	None	11
PB2	G -> A	501	167	AAG -> AAA		SNP (transition)	None	2
PB2	A -> G	515	172	GAC -> GGC	D -> G	SNP (transition)	Substitution	1
PB2	C -> T	516	172	GAC -> GAT		SNP (transition)	None	1
PB2	A -> G	526	176	ACT -> GCT	T -> A	SNP (transition)	Substitution	10
PB2	C -> T	625	209	CTG -> TTG		SNP (transition)	None	9
PB2	G -> A	684	228	AGG -> AGA		SNP (transition)	None	3
PB2	C -> T	705	235	AGC -> AGT		SNP (transition)	None	1
PB2	A -> G	708	236	TTA -> TTG		SNP (transition)	None	9
PB2	T -> C	743	248	ATA -> ACA	I -> T	SNP (transition)	Substitution	11
PB2	T -> C	918	306	ACT -> ACC		SNP (transition)	None	2
PB2	A -> G	932	311	AAT -> AGT	N -> S	SNP (transition)	Substitution	9
PB2	G -> T	943	315	GTC -> TTC	V -> F	SNP (transversion)	Substitution	5
PB2	G -> A	951	317	AAG -> AAA		SNP (transition)	None	11
PB2	A -> G	959	320	GAA -> GGA	E -> G	SNP (transition)	Substitution	4
PB2	A -> G	1162	388	ATT -> GTT	I -> V	SNP (transition)	Substitution	1
PB2	T -> C	1521	507	ACT -> ACC		SNP (transition)	None	1
PB2	A -> G	1530	510	TCA -> TCG		SNP (transition)	None	1
PB2	G -> A	1689	563	AAG -> AAA		SNP (transition)	None	5
PB2	G -> A	1749	583	GTG -> GTA		SNP (transition)	None	1
PB2	A -> G	1812	604	GAA -> GAG		SNP (transition)	None	3
PB2	A -> G	1910	637	AAG -> AGG	K -> R	SNP (transition)	Substitution	3
