# Supplementary material for: Spreading of the High-Pathogenicity Avian Influenza (H5N1) Virus of Clade 2.3.4.4b into Uruguay
Source: Viruses. 2023 Sep 11;15(9):1906. doi: 10.3390/v15091906 (PMC10536905; doi:10.3390/v15091906)
Supplement: Supplementary file 1 [file viruses-15-01906-s001.zip › Table S2.pdf]

Table S2. Sequences used for reassortment analysis showed in Figure 3. Accession numbers (Accession) for the eight avian influenza segments (Segment), country of origin (Country). the strain name (Strains) and the host species (Host) are indicated.

Accession	Segment	Country	Strain	Host
OL334651	1 (PB2)	USA	A/mallard/Alaska/AH0176840/2021(H5)	mallard; live bird surveillance
OL638152	1 (PB2)	Czech Republic	A/goose/Czech Republic/18520-2/2021(H5N1)	goose
OP269979	1 (PB2)	USA	A/Vulpes vulpes/USA	Vulpes vulpes
OP269990	1 (PB2)	USA	A/Vulpes vulpes/USA	Vulpes vulpes
OP377467	1 (PB2)	USA	A/bald eagle/Florida/W22-153B/2022(H5N1)	Haliaeetus leucocephalus
OP377533	1 (PB2)	USA	A/common eiders/Maine/W22-481B/2022(H5N1)	Somateria mollissima
OP377605	1 (PB2)	USA	A/common eiders/Maine/W22-481A/2022(H5N1)	Somateria mollissima
OP597635	1 (PB2)	Russia	A/pelican/Tumen/932-1/2021(H5N1)	pelican
OP691321	1 (PB2)	Mexico	A/Falco_rusticolus/EdoMex/CPA-19638-22/2022(H5N1)	Falco_rusticolus
OQ584581	1 (PB2)	USA	A/black vulture/South Carolina/W22-623/2022(H5N1)	Coragyps atratus
OQ632836	1 (PB2)	France	A/Mule-duck/France/21343/2021(H5N1)	Mule-duck
OQ632837	1 (PB2)	France	A/Mule-duck/France/21347/2021(H5N1)	Mule-duck
OQ632839	1 (PB2)	France	A/Mule-duck/France/21349/2021(H5N1)	Mule-duck
OQ632841	1 (PB2)	France	A/Mule-duck/France/21356/2021(H5N1)	Mule-duck
OQ632842	1 (PB2)	France	A/Pekin-duck/France/22005/2022(H5N1)	Pekin-duck
OQ730502	1 (PB2)	USA	A/harbor seal/Maine/MME-22-185Pv-ns/2022(H5N1)	Phoca vitulina
OQ733091	1 (PB2)	USA	A/striped skunk/Kansas/W23-094/2023(H5N1)	Mephitis mephitis
OQ734917	1 (PB2)	USA	A/great-tailed grackle/Kansas/W22-1223C/2022(H5N1)	Quiscalus mexicanus
OQ734954	1 (PB2)	USA	A/great-tailed grackle/Kansas/W22-1223B/2022(H5N1)	Quiscalus mexicanus
OQ734963	1 (PB2)	USA	A/great-tailed grackle/Kansas/W22-1223A/2022(H5N1)	Quiscalus mexicanus
OQ843962	1 (PB2)	USA	A/environment/Wright County/Wright-A/2022(H5N1)	NA
OQ954551	1 (PB2)	USA	A/striped skunk/Kansas/W23-175/2023(H5N1)	Mephitis mephitis
OR125139	1 (PB2)	Chile	A/chicken/Nuble/240684/2023(H5N1)	Gallus gallus
OR125144	1 (PB2)	Chile	A/Blackish oystercatcher/OHiggins/240628/2023(H5N1)	Haematopus ater
OR125167	1 (PB2)	Chile	A/Humboldt penguin/Coquimbo/239590/2023(H5N1)	Spheniscus humboldti
OR125172	1 (PB2)	Chile	A/chicken/Nuble/239136/2023(H5N1)	Gallus gallus
OR125198	1 (PB2)	Chile	A/chicken/Nuble/240155/2023(H5N1)	Gallus gallus
OR125208	1 (PB2)	Chile	A/Black Skimmer/Maule/240379/2023(H5N1)	Rynchops niger
OR125248	1 (PB2)	Chile	A/Sanderling/Arica y Parinacota/230758-1/2022(H5N1)	Calidris alba
OR125332	1 (PB2)	Chile	A/goose/Araucania/239189-1/2023(H5N1)	Anser sp.
OR136521	1 (PB2)	USA	A/Silver Pheasant/OH/OH22-20552/2022(H5N1)	Silver Pheasant; Lophura nycthemera
OR136529	1 (PB2)	USA	A/Swan/OH/OH22-22368-2/2022(H5N1)	Swan; Cygnus sp.
OR136537	1 (PB2)	USA	A/Turkey/OH/OH22-21172-1/2022(H5N1)	Turkey; Meleagris gallopavo domesticus
OR136545	1 (PB2)	USA	A/Wood Duck/OH/OH22-20551/2022(H5N1)	Wood Duck; Aix sponsa
OR136561	1 (PB2)	USA	A/chicken/OH/OH22-20542-2/2022(H5N1)	Chicken; Gallus gallus domesticus
OR136585	1 (PB2)	USA	A/chicken/OH/OH22-26275-2/2022(H5N1)	Chicken; Gallus gallus domesticus
OR136616	1 (PB2)	USA	A/goose/OH/OH22-21298/2022(H5N1)	Goose; Anser cygnoides domesticus
OR165077	1 (PB2)	USA	A/black vulture/Georgia/W22-487/2022(H5N1)	Coragyps atratus
OP221048	1 (PB2)	USA	A/bald eagle/Kansas/W22-197/2022	bald eagle
OP221292	1 (PB2)	USA	A/snow goose/Kansas/W22-199D/2022	snow goose
OP221300	1 (PB2)	USA	A/snow goose/Kansas/W22-199F/2022	snow goose
OP221317	1 (PB2)	USA	A/bald eagle/Florida/W22-191/2022	bald eagle
OP221373	1 (PB2)	USA	A/bald eagle/South Carolina/W22-205/2022	bald eagle
OP377329	1 (PB2)	USA	A/snow goose/North Dakota/N22-06/2022	snow goose
OP377345	1 (PB2)	USA	A/black vulture/Virginia/W22-499C/2022	black vulture
OP377371	1 (PB2)	USA	A/snow goose/North Dakota/N22-05/2022	snow goose
OP377387	1 (PB2)	USA	A/bald eagle/Virginia/W22-306/2022	bald eagle
OP377403	1 (PB2)	USA	A/muscovy duck/Florida/W22-306/2022	muscovy duck
OP377419	1 (PB2)	USA	A/royal tern/Florida/W22-245B/2022	royal tern
OP377509	1 (PB2)	USA	A/common tern/Maine/W22-480A/2022	common tern
OP377549	1 (PB2)	USA	A/great blue heron/Florida/W22-160/2022	great blue heron
OP377581	1 (PB2)	USA	A/black vulture/Florida/W22-167/2022	black vulture
OP377589	1 (PB2)	USA	A/lesser snow goose/North Dakota/ND-10/2022	lesser snow goose
OP377597	1 (PB2)	USA	A/snow goose/Kansas/W22-174B/2022	snow goose
OP377613	1 (PB2)	USA	A/brown pelican/North Carolina/W22-164/2022	brown pelican
OP377629	1 (PB2)	USA	A/bald eagle/North Carolina/W22-140/2022	bald eagle
OP470756	1 (PB2)	USA	A/lesser scaup/Georgia/W22-145B/2022	lesser scaup
OP470764	1 (PB2)	USA	A/lesser scaup/Georgia/W22-145D/2022	lesser scaup
OP470796	1 (PB2)	USA	A/snow goose/Kentucky/W22-092/2022	snow goose
OP950294	1 (PB2)	Venezuela	A/Pelecanus occidentalis/Venezuela/Pel3S1/2022	Pelecanus occidentalis
OQ352553	1 (PB2)	Chile	A/gray gull/Chile/C61947/2022	gray gull
OQ455434	1 (PB2)	Chile	A/Gull/CHL/227023-2/2022	Gull
OQ455439	1 (PB2)	Chile	A/Gull/CHL/227023-3/2022	Gull
OQ455444	1 (PB2)	Chile	A/Pelican/CHL/226618-2/2022	Pelican
OQ455463	1 (PB2)	Chile	A/Pelican/CHL/226924-1/2022	Pelican
OQ547317	1 (PB2)	Peru	A/Gallus gallus/Peru/AIS0547/2022	Gallus gallus
OQ547348	1 (PB2)	Peru	A/Gallus gallus/Peru/AIS0540/2022	Gallus gallus
OQ547404	1 (PB2)	Peru	A/Gallus gallus/Peru/AIS0550/2022	Gallus gallus
OQ565625	1 (PB2)	Peru	A/Pelecanus/Peru/VFAR-140/2022	Pelecanus
OQ584527	1 (PB2)	USA	A/black vulture/Georgia/W22-404A/2022	black vulture
OQ584543	1 (PB2)	USA	A/black vulture/South Carolina/W22-689B/2022	black vulture
OQ584637	1 (PB2)	USA	A/bald eagle/Kansas/W22-384/2022	bald eagle
OQ584708	1 (PB2)	USA	A/black vulture/Virginia/W22-667C/2022	black vulture
OQ584716	1 (PB2)	USA	A/black vulture/Georgia/W22-749A/2022	black vulture
OQ584732	1 (PB2)	USA	A/black vulture/Georgia/W22-404B/2022	black vulture
OQ632896	1 (PB2)	France	A/chicken/France/21328/2021	chicken
OQ683455	1 (PB2)	Colombia	A/chicken/Colombia/Bolivar/3500/2022	chicken
OQ683463	1 (PB2)	Colombia	A/chicken/Colombia/Choco/3502/2022	chicken
OQ683479	1 (PB2)	Colombia	A/chicken/Colombia/Cordoba/3499/2022	chicken
OQ683487	1 (PB2)	Colombia	A/chicken/Colombia/Magdalena/3503/2022	chicken
OQ694853	1 (PB2)	USA	A/snow goose/Kansas/W22-1143/2022	snow goose
OQ694861	1 (PB2)	USA	A/black vulture/North Carolina/W22-1078/2022	black vulture
OQ694869	1 (PB2)	USA	A/black vulture/South Carolina/W22-1112/2022	black vulture
OQ694885	1 (PB2)	USA	A/snow goose/Louisana/W22-1163/2022	snow goose
OQ694909	1 (PB2)	USA	A/Canada goose/North Carolina/W22-1055/2022	Canada goose
OQ694927	1 (PB2)	USA	A/mallard/North Carolina/W22-1114A/2022	mallard
OQ694935	1 (PB2)	USA	A/snow goose/Louisana/W22-1146A/2022	snow goose
OQ694951	1 (PB2)	USA	A/black vulture/Tennessee/W22-1150B/2022	black vulture
OQ694959	1 (PB2)	USA	A/black vulture/Tennessee/W22-1149/2022	black vulture
OQ696059	1 (PB2)	USA	A/Ross's goose/Kansas/W22-1154/2022	Ross's goose
OQ696070	1 (PB2)	USA	A/red-tailed hawk/Kansas/W22-1155/2022	red-tailed hawk
OQ732947	1 (PB2)	USA	A/black vulture/Georgia/W22-969B/2022	black vulture
OQ732963	1 (PB2)	USA	A/blue-winged teal/Minnesota/AV22-690/2022	blue-winged teal
OQ732971	1 (PB2)	USA	A/red-tailed hawk/Kentucky/W23-143/2022	red-tailed hawk
OQ732979	1 (PB2)	USA	A/blue-winged teal/Texas/UGAI22-2966/2022	blue-winged teal
OQ732987	1 (PB2)	USA	A/black vulture/Georgia/W22-933C/2022	black vulture
OQ733003	1 (PB2)	USA	A/blue-winged teal/Texas/UGAI22-3268/2022	blue-winged teal
OQ733011	1 (PB2)	USA	A/blue-winged teal/Minnesota/UGAI22-3620/2022	blue-winged teal
OQ733019	1 (PB2)	USA	A/blue-winged teal/Texas/UGAI22-3250/2022	blue-winged teal
OQ733027	1 (PB2)	USA	A/bald eagle/North Carolina/W23-142A/2023	bald eagle
OQ733035	1 (PB2)	USA	A/blue-winged teal/Texas/UGAI22-3189/2022	blue-winged teal
OQ733067	1 (PB2)	USA	A/black vulture/Georgia/W22-972A/2022	black vulture
OQ733075	1 (PB2)	USA	A/bald eagle/South Carolina/W23-142A/2023	bald eagle
OQ733083	1 (PB2)	USA	A/blue-winged teal/Texas/UGAI22-3226/2022	blue-winged teal
OQ733099	1 (PB2)	USA	A/bald eagle/Virginia/W22-101/2023	bald eagle
OQ733115	1 (PB2)	USA	A/blue-winged teal/Texas/UGAI22-2961/2022	blue-winged teal
OQ733131	1 (PB2)	USA	A/blue-winged teal/Minnesota/UGAI22-3611/2022	blue-winged teal

OQ733147	1 (PB2)	USA	A/Canada goose/Virginia/W22-773/2022	Canada goose
OQ733155	1 (PB2)	USA	A/blue-winged teal/Minnesota/AV22-680/2022	blue-winged teal
OQ734885	1 (PB2)	USA	A/red-tailed hawk/Kansas/W22-1226/2022	red-tailed hawk
OQ734909	1 (PB2)	USA	A/Cooper's hawk/Kansas/W22-1206/2022	Cooper's hawk
OQ734925	1 (PB2)	USA	A/brown pelican/North Carolina/W23-019/2022	brown pelican
OQ734944	1 (PB2)	USA	A/herring gull/North Carolina/W1215B/2022	herring gull
OQ734971	1 (PB2)	USA	A/black vulture/North Carolina/W22-1213/2022	black vulture
OQ734979	1 (PB2)	USA	A/bald eagle/Virginia/W22-1222/2022	bald eagle
OQ737776	1 (PB2)	USA	A/great black-backed gull/North Carolina/W22-1215A/2022	great black-backed gull
OQ851654	1 (PB2)	USA	A/Pekin duck/California/T2202390/2022	Pekin duck
OQ954543	1 (PB2)	USA	A/black vulture/Louisiana/W23-166/2023	black vulture
OQ982395	1 (PB2)	USA	A/mallard duck/Minnesota/UGAI22-3846/2022	mallard duck
OQ982411	1 (PB2)	USA	A/blue-winged teal/Louisiana/UGAI22-3889/2022	blue-winged teal
OQ982419	1 (PB2)	USA	A/blue-winged teal/Minnesota/UGAI22-3834/2022	blue-winged teal
OQ982427	1 (PB2)	USA	A/blue-winged teal/Louisiana/UGAI22-3875/2022	blue-winged teal
MW873715	1 (PB2)	USA	A/mallard/Alaska/AK20-526/2020(H5N2)	mallard
MZ564969	1 (PB2)	Mexico	A/chicken/Jalisco/CPA-03604-19/2020(H5N2)	chicken
OL461745	1 (PB2)	USA	A/mallard/Oregon/AH0192864/2021(H5N2)	mallard; live bird surveillance
OM965818	1 (PB2)	USA	A/Sanderling/Delaware/518/2021(H5N2)	Calidris alba
OQ366643	1 (PB2)	USA	A/Ruddy Turnstone/DE/495/2022	Ruddy Turnstone
OQ366658	1 (PB2)	USA	A/Ruddy Turnstone/DE/464/2022	Ruddy Turnstone
MW875337	1 (PB2)	USA	A/Red knot/Delaware Bay/404/2020(H5N3)	Calidris canutus
OL514115	1 (PB2)	USA	A/mallard/Vermont/AH0188057/2021(H5N3)	mallard; live bird surveillance
OL514123	1 (PB2)	USA	A/mallard/Vermont/AH0188065/2021(H5N3)	mallard; live bird surveillance
OL514155	1 (PB2)	USA	A/mallard/New York/AH0179308/2021(H5N3)	mallard; live bird surveillance
OL514218	1 (PB2)	USA	A/mallard/Vermont/AH0188140/2021(H5N3)	mallard; live bird surveillance
OL539333	1 (PB2)	USA	A/mallard/Maine/AH0179819/2021(H5N3)	mallard; live bird surveillance
OL539626	1 (PB2)	USA	A/mallard/New York/AH0179389/2021(H5N3)	mallard; live bird surveillance
OL583750	1 (PB2)	USA	A/mallard/New Hampshire/AH0190828/2021(H5N3)	mallard; live bird surveillance
OL422845	1 (PB2)	USA	A/mallard/New York/AH0179249/2021(H5N4)	mallard; live bird surveillance
MW875202	1 (PB2)	USA	A/ruddy turnstone/Delaware Bay/374/2020(H5N9)	Arenaria interpres
MW875333	1 (PB2)	USA	A/ruddy turnstone/Delaware Bay/386/2020(H5N9)	Arenaria interpres
OL638151	2 (PB1)	Czech Republic	A/goose/Czech Republic/18520-2/2021(H5N1)	goose
OP269961	2 (PB1)	USA	A/Vulpes vulpes/USA	Vulpes vulpes
OP270004	2 (PB1)	USA	A/Vulpes vulpes/USA	Vulpes vulpes
OP270017	2 (PB1)	USA	A/Vulpes vulpes/USA	Vulpes vulpes
OP377532	2 (PB1)	USA	A/common eiders/Maine/W22-481B/2022(H5N1)	Somateria mollissima
OP499859	2 (PB1)	USA	A/Lesser scaup/MD/-LC-EESC-024/2022(H5N1)	Aythya affinis
OP597617	2 (PB1)	Russia	A/common teal/Chelyabinsk/1379-1/2021(H5N1)	common teal
OP597625	2 (PB1)	Russia	A/goose/Chelyabinsk/1341-3/2021(H5N1)	goose
OP597641	2 (PB1)	Russia	A/pelican/Tumen/932-1/2021(H5N1)	pelican
OP691322	2 (PB1)	Mexico	A/Falco_rusticolus/EdoMex/CPA-19638-22/2022(H5N1)	Falco_rusticolus
OQ584502	2 (PB1)	USA	A/black vulture/Georgia/W22-723A/2022(H5N1)	Coragyps atratus
OQ584580	2 (PB1)	USA	A/black vulture/South Carolina/W22-623/2022(H5N1)	Coragyps atratus
OQ632843	2 (PB1)	France	A/Pekin-duck/France/22005/2022(H5N1)	Pekin-duck
OQ734916	2 (PB1)	USA	A/great-tailed grackle/Kansas/W22-1223C/2022(H5N1)	Quiscalus mexicanus
OQ734955	2 (PB1)	USA	A/great-tailed grackle/Kansas/W22-1223B/2022(H5N1)	Quiscalus mexicanus
OQ734962	2 (PB1)	USA	A/great-tailed grackle/Kansas/W22-1223A/2022(H5N1)	Quiscalus mexicanus
OR125153	2 (PB1)	Chile	A/South American tern/Maule/238507/2023(H5N1)	Sterna hirundinacea
OR125173	2 (PB1)	Chile	A/chicken/Nuble/239136/2023(H5N1)	Gallus gallus
OR125199	2 (PB1)	Chile	A/chicken/Nuble/240155/2023(H5N1)	Gallus gallus
OR125267	2 (PB1)	Chile	A/Pelican/Valparaiso/233091-2/2023(H5N1)	Pelecanus sp.
OR125291	2 (PB1)	Chile	A/Pelican/Valparaiso/233450-1/2023(H5N1)	Pelecanus sp.
OR125311	2 (PB1)	Chile	A/Pelican/Atacama/229424-2/2022(H5N1)	Pelecanus sp.
OR125317	2 (PB1)	Chile	A/chicken/OHiggins/241252-6/2023(H5N1)	Gallus gallus
OR125333	2 (PB1)	Chile	A/goose/Araucania/239189-1/2023(H5N1)	Anser sp.
OR125360	2 (PB1)	Chile	A/Chiloe wigeon/OHiggins/240893-2/2023(H5N1)	Mareca sibilatrix
OR125382	2 (PB1)	Chile	A/chicken/Nuble/241681-1/2023(H5N1)	Gallus gallus
OR125438	2 (PB1)	Chile	A/Domestic duck/Maule/240466-1/2023(H5N1)	Anas platyrhynchos domesticus
OR136530	2 (PB1)	USA	A/Swan/OH/OH22-22368-2/2022(H5N1)	Swan; Cygnus sp.
OR136562	2 (PB1)	USA	A/chicken/OH/OH22-20542-2/2022(H5N1)	Chicken; Gallus gallus domesticus
OR136594	2 (PB1)	USA	A/chicken/OH/OH22-26275-3/2022(H5N1)	Chicken; Gallus gallus domesticus
OR136602	2 (PB1)	USA	A/chicken/OH/OH22-7075/2022(H5N1)	Chicken; Gallus gallus domesticus
OR136615	2 (PB1)	USA	A/goose/OH/OH22-21298/2022(H5N1)	Goose; Anser cygnoides domesticus
OR165028	2 (PB1)	USA	A/common eider/Massachusetts/W22-438D/2022(H5N1)	Somateria mollissima
OR165068	2 (PB1)	USA	A/black vulture/Georgia/W22-406/2022(H5N1)	Coragyps atratus
OR165076	2 (PB1)	USA	A/black vulture/Georgia/W22-487/2022(H5N1)	Coragyps atratus
OP221291	2 (PB1)	USA	A/snow goose/Kansas/W22-199D/2022	snow goose
OP221316	2 (PB1)	USA	A/bald eagle/Florida/W22-191/2022	bald eagle
OP221333	2 (PB1)	USA	A/bald eagle/Florida/W22-195/2022	bald eagle
OP221348	2 (PB1)	USA	A/American pelican/Kansas/W22-200/2022	American pelican
OP221372	2 (PB1)	USA	A/bald eagle/South Carolina/W22-205/2022	bald eagle
OP222197	2 (PB1)	USA	A/bald eagle/Florida/W22-189/2022	bald eagle
OP377328	2 (PB1)	USA	A/snow goose/North Dakota/N22-06/2022	snow goose
OP377336	2 (PB1)	USA	A/snow goose/North Dakota/N22-04/2022	snow goose
OP377344	2 (PB1)	USA	A/black vulture/Virginia/W22-499C/2022	black vulture
OP377402	2 (PB1)	USA	A/muscovy duck/Florida/W22-306/2022	muscovy duck
OP377410	2 (PB1)	USA	A/bald eagle/North Carolina/W22-186/2022	bald eagle
OP377426	2 (PB1)	USA	A/gull/Florida/W22-162/2022	gull
OP377508	2 (PB1)	USA	A/common tern/Maine/W22-480A/2022	common tern
OP377524	2 (PB1)	USA	A/snow goose/Kansas/W22-260/2022	snow goose
OP377564	2 (PB1)	USA	A/black vulture/North Carolina/W22-367C/2022	black vulture
OP377588	2 (PB1)	USA	A/lesser snow goose/North Dakota/ND-10/2022	lesser snow goose
OP377628	2 (PB1)	USA	A/bald eagle/North Carolina/W22-140/2022	bald eagle
OP377636	2 (PB1)	USA	A/hooded merganser/Florida/W22-154/2022	hooded merganser
OP470763	2 (PB1)	USA	A/lesser scaup/Georgia/W22-145D/2022	lesser scaup
OP470771	2 (PB1)	USA	A/lesser scaup/Georgia/W22-143/2022	lesser scaup
OP470787	2 (PB1)	USA	A/lesser scaup/Georgia/W22-145E/2022	lesser scaup
OP470795	2 (PB1)	USA	A/snow goose/Kentucky/W22-092/2022	snow goose
OP950295	2 (PB1)	Venezuela	A/Pelecanus occidentalis/Venezuela/Pel3S2/2022	Pelecanus occidentalis
OQ352546	2 (PB1)	Chile	A/black skimmer/Chile/C61962/2022	black skimmer
OQ352554	2 (PB1)	Chile	A/gray gull/Chile/C61947/2022	gray gull
OQ455435	2 (PB1)	Chile	A/Gull/CHL/227023-2/2022	Gull
OQ455440	2 (PB1)	Chile	A/Gull/CHL/227023-3/2022	Gull
OQ455445	2 (PB1)	Chile	A/Pelican/CHL/226618-2/2022	Pelican
OQ547341	2 (PB1)	Peru	A/Gallus gallus/Peru/AIS0539/2022	Gallus gallus
OQ547389	2 (PB1)	Peru	A/Gallus gallus/Peru/AIS0548/2022	Gallus gallus
OQ565626	2 (PB1)	Peru	A/Pelecanus/Peru/VFAR-140/2022	Pelecanus
OQ584526	2 (PB1)	USA	A/black vulture/Georgia/W22-404A/2022	black vulture
OQ584542	2 (PB1)	USA	A/black vulture/South Carolina/W22-689B/2022	black vulture
OQ584636	2 (PB1)	USA	A/bald eagle/Kansas/W22-384/2022	bald eagle
OQ584684	2 (PB1)	USA	A/black vulture/Georgia/W22-749B/2022	black vulture
OQ584707	2 (PB1)	USA	A/black vulture/Virginia/W22-667C/2022	black vulture
OQ584731	2 (PB1)	USA	A/black vulture/Georgia/W22-404B/2022	black vulture
OQ584739	2 (PB1)	USA	A/black vulture/Georgia/W22-395/2022	black vulture
OQ632897	2 (PB1)	France	A/chicken/France/21328/2021	chicken
OQ683456	2 (PB1)	Colombia	A/chicken/Colombia/Bolivar/3500/2022	chicken
OQ683480	2 (PB1)	Colombia	A/chicken/Colombia/Cordoba/3499/2022	chicken

OQ683488	2 (PB1)	Colombia	A/chicken/Colombia/Magdalena/3503/2022	chicken
OQ683496	2 (PB1)	Colombia	A/wild duck/Colombia/Choco/3501/2022	wild duck
OQ694852	2 (PB1)	USA	A/snow goose/Kansas/W22-1143/2022	snow goose
OQ694860	2 (PB1)	USA	A/black vulture/North Carolina/W22-1078/2022	black vulture
OQ694868	2 (PB1)	USA	A/black vulture/South Carolina/W22-1112/2022	black vulture
OQ694876	2 (PB1)	USA	A/black vulture/South Carolina/W22-1080B/2022	black vulture
OQ694884	2 (PB1)	USA	A/snow goose/Louisana/W22-1163/2022	snow goose
OQ694892	2 (PB1)	USA	A/black vulture/Georgia/W22-1049/2022	black vulture
OQ694926	2 (PB1)	USA	A/mallard/North Carolina/W22-1114A/2022	mallard
OQ694934	2 (PB1)	USA	A/snow goose/Louisana/W22-1146A/2022	snow goose
OQ694958	2 (PB1)	USA	A/black vulture/Tennessee/W22-1149/2022	black vulture
OQ696058	2 (PB1)	USA	A/Ross's goose/Kansas/W22-1154/2022	Ross's goose
OQ732946	2 (PB1)	USA	A/black vulture/Georgia/W22-969B/2022	black vulture
OQ732962	2 (PB1)	USA	A/blue-winged teal/Minnesota/AV22-690/2022	blue-winged teal
OQ732970	2 (PB1)	USA	A/red-tailed hawk/Kentucky/W23-143/2022	red-tailed hawk
OQ732978	2 (PB1)	USA	A/blue-winged teal/Texas/UGAI22-2966/2022	blue-winged teal
OQ732986	2 (PB1)	USA	A/black vulture/Georgia/W22-933C/2022	black vulture
OQ733002	2 (PB1)	USA	A/blue-winged teal/Texas/UGAI22-3268/2022	blue-winged teal
OQ733010	2 (PB1)	USA	A/blue-winged teal/Minnesota/UGAI22-3620/2022	blue-winged teal
OQ733034	2 (PB1)	USA	A/blue-winged teal/Texas/UGAI22-3189/2022	blue-winged teal
OQ733066	2 (PB1)	USA	A/black vulture/Georgia/W22-972A/2022	black vulture
OQ733074	2 (PB1)	USA	A/bald eagle/South Carolina/W23-142A/2023	bald eagle
OQ733082	2 (PB1)	USA	A/blue-winged teal/Texas/UGAI22-3226/2022	blue-winged teal
OQ733114	2 (PB1)	USA	A/blue-winged teal/Texas/UGAI22-2961/2022	blue-winged teal
OQ733122	2 (PB1)	USA	A/black vulture/Georgia/W22-1056A/2022	black vulture
OQ733154	2 (PB1)	USA	A/blue-winged teal/Minnesota/AV22-680/2022	blue-winged teal
OQ734892	2 (PB1)	USA	A/bald eagle/Tennessee/W23-003/2022	bald eagle
OQ734900	2 (PB1)	USA	A/American green-winged teal/Texas/UGAI22-3462/2022	American green-winged teal
OQ734908	2 (PB1)	USA	A/Cooper's hawk/Kansas/W22-1206/2022	Cooper's hawk
OQ734924	2 (PB1)	USA	A/brown pelican/North Carolina/W23-019/2022	brown pelican
OQ734943	2 (PB1)	USA	A/herring gull/North Carolina/W1215B/2022	herring gull
OQ734970	2 (PB1)	USA	A/black vulture/North Carolina/W22-1213/2022	black vulture
OQ734978	2 (PB1)	USA	A/bald eagle/Virginia/W22-1222/2022	bald eagle
OQ737775	2 (PB1)	USA	A/great black-backed gull/North Carolina/W22-1215A/2022	great black-backed gull
OQ747878	2 (PB1)	Peru	A/Peruvian_pelican/Peru/A074/2022	Peruvian_pelican
OQ747883	2 (PB1)	Peru	A/Western_barn_owl/Peru/A293/2022	Western_barn_owl
OQ851653	2 (PB1)	USA	A/Pekin duck/California/T2202390/2022	Pekin duck
OQ954534	2 (PB1)	USA	A/bald eagle/South Carolina/W23-201B/2023	bald eagle
OQ982402	2 (PB1)	USA	A/bald eagle/North Carolina/W23-012/2022	bald eagle
OQ982410	2 (PB1)	USA	A/blue-winged teal/Louisiana/UGAI22-3889/2022	blue-winged teal
OQ982418	2 (PB1)	USA	A/blue-winged teal/Minnesota/UGAI22-3834/2022	blue-winged teal
OQ982426	2 (PB1)	USA	A/blue-winged teal/Louisiana/UGAI22-3875/2022	blue-winged teal
OL636397	3 (PA)	Czech Republic	A/goose/Czech Republic/18520-1/2021(H5N1)	goose
OP269953	3 (PA)	USA	A/Vulpes vulpes/USA	Vulpes vulpes
OP377531	3 (PA)	USA	A/common eiders/Maine/W22-481B/2022(H5N1)	Somateria mollissima
OP499860	3 (PA)	USA	A/Lesser scaup/MD/-LC-EESC-024/2022(H5N1)	Aythya affinis
OP597616	3 (PA)	Russia	A/common teal/Chelyabinsk/1379-1/2021(H5N1)	common teal
OP597624	3 (PA)	Russia	A/goose/Chelyabinsk/1341-3/2021(H5N1)	goose
OP597632	3 (PA)	Russia	A/pelican/Tumen/1032-1/2021(H5N1)	pelican
OP691323	3 (PA)	Mexico	A/Falco_rusticolus/EdoMex/CPA-19638-22/2022(H5N1)	Falco_rusticolus
OP698127	3 (PA)	USA	A/bottlenose dolphin/Florida/UFTt2203/2022(H5N1)	Tursiops truncatus
OQ584501	3 (PA)	USA	A/black vulture/Georgia/W22-723A/2022(H5N1)	Coragyps atratus
OQ595414	3 (PA)	USA	A/bald eagle/North Carolina/W22-729/2022(H5N1)	Haliaeetus leucocephalus
OQ730468	3 (PA)	USA	A/harbor seal/Maine/MME-22-150Pv-ns/2022(H5N1)	Phoca vitulina
OQ730492	3 (PA)	USA	A/harbor seal/Maine/MME-22-117Pv-ns/2022(H5N1)	Phoca vitulina
OQ732939	3 (PA)	USA	A/harbor seal/Maine/MME-22-147Pv-L/2022(H5N1)	Phoca vitulina
OQ733089	3 (PA)	USA	A/striped skunk/Kansas/W23-094/2023(H5N1)	Mephitis mephitis
OQ734915	3 (PA)	USA	A/great-tailed grackle/Kansas/W22-1223C/2022(H5N1)	Quiscalus mexicanus
OQ734953	3 (PA)	USA	A/great-tailed grackle/Kansas/W22-1223B/2022(H5N1)	Quiscalus mexicanus
OQ734961	3 (PA)	USA	A/great-tailed grackle/Kansas/W22-1223A/2022(H5N1)	Quiscalus mexicanus
OQ954549	3 (PA)	USA	A/striped skunk/Kansas/W23-175/2023(H5N1)	Mephitis mephitis
OR125140	3 (PA)	Chile	A/chicken/Nuble/240684/2023(H5N1)	Gallus gallus
OR125187	3 (PA)	Chile	A/Sanderling/Arica y Parinacota/240265/2023(H5N1)	Calidris alba
OR125200	3 (PA)	Chile	A/chicken/Nuble/240155/2023(H5N1)	Gallus gallus
OR125243	3 (PA)	Chile	A/chicken/OHiggins/241252-3/2023(H5N1)	Gallus gallus
OR125268	3 (PA)	Chile	A/Pelican/Valparaiso/233091-2/2023(H5N1)	Pelecanus sp.
OR125439	3 (PA)	Chile	A/Domestic duck/Maule/240466-1/2023(H5N1)	Anas platyrhynchos domesticus
OR125476	3 (PA)	Chile	A/Humboldt penguin/Tarapaca/238744-2/2023(H5N1)	Spheniscus humboldti
OR136523	3 (PA)	USA	A/Silver Pheasant/OH/OH22-20552/2022(H5N1)	Silver Pheasant; Lophura nycthemera
OR136531	3 (PA)	USA	A/Swan/OH/OH22-22368-2/2022(H5N1)	Swan; Cygnus sp.
OR136547	3 (PA)	USA	A/Wood Duck/OH/OH22-20551/2022(H5N1)	Wood Duck; Aix sponsa
OR136555	3 (PA)	USA	A/chicken/OH/OH22-20542-1/2022(H5N1)	Chicken; Gallus gallus domesticus
OR136571	3 (PA)	USA	A/chicken/OH/OH22-21172-2/2022(H5N1)	Chicken; Gallus gallus domesticus
OR136579	3 (PA)	USA	A/chicken/OH/OH22-26275-1/2022(H5N1)	Chicken; Gallus gallus domesticus
OR136587	3 (PA)	USA	A/chicken/OH/OH22-26275-2/2022(H5N1)	Chicken; Gallus gallus domesticus
OR136595	3 (PA)	USA	A/chicken/OH/OH22-26275-3/2022(H5N1)	Chicken; Gallus gallus domesticus
OR136603	3 (PA)	USA	A/chicken/OH/OH22-7075/2022(H5N1)	Chicken; Gallus gallus domesticus
OR136614	3 (PA)	USA	A/goose/OH/OH22-21298/2022(H5N1)	Goose; Anser cygnoides domesticus
OR165067	3 (PA)	USA	A/black vulture/Georgia/W22-406/2022(H5N1)	Coragyps atratus
OR165075	3 (PA)	USA	A/black vulture/Georgia/W22-487/2022(H5N1)	Coragyps atratus
OP221046	3 (PA)	USA	A/bald eagle/Kansas/W22-197/2022	bald eagle
OP221290	3 (PA)	USA	A/snow goose/Kansas/W22-199D/2022	snow goose
OP221298	3 (PA)	USA	A/snow goose/Kansas/W22-199F/2022	snow goose
OP221306	3 (PA)	USA	A/bald eagle/Georgia/W22-194B/2022	bald eagle
OP221332	3 (PA)	USA	A/bald eagle/Florida/W22-195/2022	bald eagle
OP221355	3 (PA)	USA	A/snow goose/Kansas/W22-199A/2022	snow goose
OP221387	3 (PA)	USA	A/bald eagle/Georgia/W22-194A/2022	bald eagle
OP222198	3 (PA)	USA	A/American pelican/Kansas/W22-200/2022	American pelican
OP377335	3 (PA)	USA	A/snow goose/North Dakota/N22-04/2022	snow goose
OP377401	3 (PA)	USA	A/muscovy duck/Florida/W22-306/2022	muscovy duck
OP377417	3 (PA)	USA	A/royal tern/Florida/W22-245B/2022	royal tern
OP377425	3 (PA)	USA	A/gull/Florida/W22-162/2022	gull
OP377441	3 (PA)	USA	A/bald eagle/Florida/W22-134-OP/2022	bald eagle
OP377507	3 (PA)	USA	A/common tern/Maine/W22-480A/2022	common tern
OP377547	3 (PA)	USA	A/great blue heron/Florida/W22-160/2022	great blue heron
OP377563	3 (PA)	USA	A/black vulture/North Carolina/W22-367C/2022	black vulture
OP377587	3 (PA)	USA	A/lesser snow goose/North Dakota/ND-10/2022	lesser snow goose
OP377611	3 (PA)	USA	A/brown pelican/North Carolina/W22-164/2022	brown pelican
OP377627	3 (PA)	USA	A/bald eagle/North Carolina/W22-140/2022	bald eagle
OP377635	3 (PA)	USA	A/hooded merganser/Florida/W22-154/2022	hooded merganser
OP470754	3 (PA)	USA	A/lesser scaup/Georgia/W22-145B/2022	lesser scaup
OP950296	3 (PA)	Venezuela	A/Pelecanus occidentalis/Venezuela/Pel3S3/2022	Pelecanus occidentalis
OP950304	3 (PA)	Venezuela	A/Pelecanus occidentalis/Venezuela/Pel4S3/2022	Pelecanus occidentalis
OQ352540	3 (PA)	Chile	A/Peruvian pelican/Chile/C61740/2022	Peruvian pelican
OQ352547	3 (PA)	Chile	A/black skimmer/Chile/C61962/2022	black skimmer
OQ455405	3 (PA)	Chile	A/Pelican/CHL/226958-1/2022	Pelican
OQ455432	3 (PA)	Chile	A/Pelican/CHL/226618-1/2022	Pelican
OQ455441	3 (PA)	Chile	A/Gull/CHL/227023-3/2022	Gull

OQ455446	3 (PA)	Chile	A/Pelican/CHL/226618-2/2022	Pelican
OQ455465	3 (PA)	Chile	A/Pelican/CHL/226924-1/2022	Pelican
OQ547334	3 (PA)	Peru	A/Pelecanus thagus/Peru/AIS0541/2022	Pelecanus thagus
OQ547358	3 (PA)	Peru	A/Gallus gallus/Peru/AIS0542/2022	Gallus gallus
OQ547374	3 (PA)	Peru	A/Gallus gallus/Peru/AIS0544/2022	Gallus gallus
OQ547390	3 (PA)	Peru	A/Gallus gallus/Peru/AIS0548/2022	Gallus gallus
OQ565627	3 (PA)	Peru	A/Pelecanus/Peru/VFAR-140/2022	Pelecanus
OQ584509	3 (PA)	USA	A/black vulture/Georgia/W22-769/2022	black vulture
OQ584517	3 (PA)	USA	A/black vulture/Georgia/W22-722B/2022	black vulture
OQ584549	3 (PA)	USA	A/black vulture/Georgia/W22-675B/2022	black vulture
OQ584557	3 (PA)	USA	A/black vulture/Georgia/W22-619A/2022	black vulture
OQ584611	3 (PA)	USA	A/black vulture/Georgia/W22-722C/2022	black vulture
OQ584714	3 (PA)	USA	A/black vulture/Georgia/W22-749A/2022	black vulture
OQ584730	3 (PA)	USA	A/black vulture/Georgia/W22-404B/2022	black vulture
OQ632898	3 (PA)	France	A/chicken/France/21328/2021	chicken
OQ683457	3 (PA)	Colombia	A/chicken/Colombia/Bolivar/3500/2022	chicken
OQ683473	3 (PA)	Colombia	A/chicken/Colombia/Choco/3504/2022	chicken
OQ683481	3 (PA)	Colombia	A/chicken/Colombia/Cordoba/3499/2022	chicken
OQ683489	3 (PA)	Colombia	A/chicken/Colombia/Magdalena/3503/2022	chicken
OQ694811	3 (PA)	USA	A/black vulture/Georgia/W22-1048/2022	black vulture
OQ694851	3 (PA)	USA	A/snow goose/Kansas/W22-1143/2022	snow goose
OQ694859	3 (PA)	USA	A/black vulture/North Carolina/W22-1078/2022	black vulture
OQ694867	3 (PA)	USA	A/black vulture/South Carolina/W22-1112/2022	black vulture
OQ694891	3 (PA)	USA	A/black vulture/Georgia/W22-1049/2022	black vulture
OQ694899	3 (PA)	USA	A/black vulture/Georgia/W22-1057B/2022	black vulture
OQ694907	3 (PA)	USA	A/Canada goose/North Carolina/W22-1055/2022	Canada goose
OQ694917	3 (PA)	USA	A/black vulture/Georgia/W22-1057D/2022	black vulture
OQ694925	3 (PA)	USA	A/mallard/North Carolina/W22-1114A/2022	mallard
OQ694933	3 (PA)	USA	A/snow goose/Louisana/W22-1146A/2022	snow goose
OQ696057	3 (PA)	USA	A/Ross's goose/Kansas/W22-1154/2022	Ross's goose
OQ696068	3 (PA)	USA	A/red-tailed hawk/Kansas/W22-1155/2022	red-tailed hawk
OQ732953	3 (PA)	USA	A/black vulture/South Carolina/W22-1080A/2022	black vulture
OQ732961	3 (PA)	USA	A/blue-winged teal/Minnesota/AV22-690/2022	blue-winged teal
OQ732969	3 (PA)	USA	A/red-tailed hawk/Kentucky/W23-143/2022	red-tailed hawk
OQ732977	3 (PA)	USA	A/blue-winged teal/Texas/UGAI22-2966/2022	blue-winged teal
OQ732985	3 (PA)	USA	A/black vulture/Georgia/W22-933C/2022	black vulture
OQ733001	3 (PA)	USA	A/blue-winged teal/Texas/UGAI22-3268/2022	blue-winged teal
OQ733009	3 (PA)	USA	A/blue-winged teal/Minnesota/UGAI22-3620/2022	blue-winged teal
OQ733049	3 (PA)	USA	A/black vulture/North Carolina/W22-1079C/2022	black vulture
OQ733065	3 (PA)	USA	A/black vulture/Georgia/W22-972A/2022	black vulture
OQ733073	3 (PA)	USA	A/bald eagle/South Carolina/W23-142A/2023	bald eagle
OQ733081	3 (PA)	USA	A/blue-winged teal/Texas/UGAI22-3226/2022	blue-winged teal
OQ733113	3 (PA)	USA	A/blue-winged teal/Texas/UGAI22-2961/2022	blue-winged teal
OQ733129	3 (PA)	USA	A/blue-winged teal/Minnesota/UGAI22-3611/2022	blue-winged teal
OQ733137	3 (PA)	USA	A/black vulture/Louisiana/W23-118/2023	black vulture
OQ733153	3 (PA)	USA	A/blue-winged teal/Minnesota/AV22-680/2022	blue-winged teal
OQ734883	3 (PA)	USA	A/red-tailed hawk/Kansas/W22-1226/2022	red-tailed hawk
OQ734891	3 (PA)	USA	A/bald eagle/Tennessee/W23-003/2022	bald eagle
OQ734899	3 (PA)	USA	A/American green-winged teal/Texas/UGAI22-3462/2022	American green-winged teal
OQ734907	3 (PA)	USA	A/Cooper's hawk/Kansas/W22-1206/2022	Cooper's hawk
OQ734923	3 (PA)	USA	A/brown pelican/North Carolina/W23-019/2022	brown pelican
OQ734931	3 (PA)	USA	A/double-crested cormorant/North Carolina/W22-1215C/2022	double-crested cormorant
OQ734942	3 (PA)	USA	A/herring gull/North Carolina/W1215B/2022	herring gull
OQ734969	3 (PA)	USA	A/black vulture/North Carolina/W22-1213/2022	black vulture
OQ747873	3 (PA)	Peru	A/Peruvian_pelican/Peru/A074/2022	Peruvian_pelican
OQ851652	3 (PA)	USA	A/Pekin duck/California/T2202390/2022	Pekin duck
OQ982401	3 (PA)	USA	A/bald eagle/North Carolina/W23-012/2022	bald eagle
OQ982409	3 (PA)	USA	A/blue-winged teal/Louisiana/UGAI22-3889/2022	blue-winged teal
OQ982425	3 (PA)	USA	A/blue-winged teal/Louisiana/UGAI22-3875/2022	blue-winged teal
OP377461	4 (HA)	USA	A/bald eagle/Florida/W22-153B/2022(H5N1)	Haliaeetus leucocephalus
OP499861	4 (HA)	USA	A/Lesser scaup/MD/-LC-EESC-024/2022(H5N1)	Aythya affinis
OQ428779	4 (HA)	USA	A/Anas platyrhynchos/AZ/S1-AZMCP12122022-S4/2022(H5N1)	Anas platyrhynchos
OQ584560	4 (HA)	USA	A/bald eagle/North Carolina/W22-729/2022(H5N1)	Haliaeetus leucocephalus
OQ584575	4 (HA)	USA	A/black vulture/South Carolina/W22-623/2022(H5N1)	Coragyps atratus
OQ584694	4 (HA)	USA	A/black vulture/Georgia/W22-619B/2022(H5N1)	Coragyps atratus
OQ600260	4 (HA)	USA	A/black vulture/Georgia/W22-723A/2022(H5N1)	Coragyps atratus
OP221041	4 (HA)	USA	A/bald eagle/Kansas/W22-197/2022	bald eagle
OP221285	4 (HA)	USA	A/snow goose/Kansas/W22-199D/2022	snow goose
OP221301	4 (HA)	USA	A/bald eagle/Georgia/W22-194B/2022	bald eagle
OP221311	4 (HA)	USA	A/bald eagle/Florida/W22-191/2022	bald eagle
OP221327	4 (HA)	USA	A/bald eagle/Florida/W22-195/2022	bald eagle
OP221335	4 (HA)	USA	A/bald eagle/Georgia/W22-202/2022	bald eagle
OP221343	4 (HA)	USA	A/American pelican/Kansas/W22-200/2022	American pelican
OP221350	4 (HA)	USA	A/snow goose/Kansas/W22-199A/2022	snow goose
OP377322	4 (HA)	USA	A/snow goose/North Dakota/N22-06/2022	snow goose
OP377330	4 (HA)	USA	A/snow goose/North Dakota/N22-04/2022	snow goose
OP377338	4 (HA)	USA	A/black vulture/Virginia/W22-499C/2022	black vulture
OP377380	4 (HA)	USA	A/bald eagle/Virginia/W22-306/2022	bald eagle
OP377396	4 (HA)	USA	A/muscovy duck/Florida/W22-306/2022	muscovy duck
OP377412	4 (HA)	USA	A/royal tern/Florida/W22-245B/2022	royal tern
OP377420	4 (HA)	USA	A/gull/Florida/W22-162/2022	gull
OP377445	4 (HA)	USA	A/bald eagle/North Carolina/W22-229/2022	bald eagle
OP377453	4 (HA)	USA	A/black vulture/Florida/W22-168/2022	black vulture
OP377486	4 (HA)	USA	A/black vulture/Florida/W22-161/2022	black vulture
OP377510	4 (HA)	USA	A/great horned owl/Flordia/W22-163A/2022	great horned owl
OP377534	4 (HA)	USA	A/bald eagle/Florida/W22-153A/2022	bald eagle
OP377542	4 (HA)	USA	A/great blue heron/Florida/W22-160/2022	great blue heron
OP377550	4 (HA)	USA	A/common tern/Maine/W22-480B/2022	common tern
OP377558	4 (HA)	USA	A/black vulture/North Carolina/W22-367C/2022	black vulture
OP377574	4 (HA)	USA	A/black vulture/Florida/W22-167/2022	black vulture
OP377582	4 (HA)	USA	A/lesser snow goose/North Dakota/ND-10/2022	lesser snow goose
OP377590	4 (HA)	USA	A/snow goose/Kansas/W22-174B/2022	snow goose
OP377606	4 (HA)	USA	A/brown pelican/North Carolina/W22-164/2022	brown pelican
OP377614	4 (HA)	USA	A/royal tern/Florida/W22-245A/2022	royal tern
OP377622	4 (HA)	USA	A/bald eagle/North Carolina/W22-140/2022	bald eagle
OP377630	4 (HA)	USA	A/hooded merganser/Florida/W22-154/2022	hooded merganser
OP470716	4 (HA)	USA	A/lesser scaup/Georgia/W22-145C/2022	lesser scaup
OP470733	4 (HA)	USA	A/bald eagle/Florida/W22-134-CL/2022	bald eagle
OP470741	4 (HA)	USA	A/bald eagle/FL/W22-114/2022	bald eagle
OP470749	4 (HA)	USA	A/lesser scaup/Georgia/W22-145B/2022	lesser scaup
OP470757	4 (HA)	USA	A/lesser scaup/Georgia/W22-145D/2022	lesser scaup
OP470765	4 (HA)	USA	A/lesser scaup/Georgia/W22-143/2022	lesser scaup
OP470781	4 (HA)	USA	A/lesser scaup/Georgia/W22-145E/2022	lesser scaup
OP470789	4 (HA)	USA	A/snow goose/Kentucky/W22-092/2022	snow goose
OP470804	4 (HA)	USA	A/bald eagle/FL/W22-114/2022	bald eagle
OP499866	4 (HA)	USA	A/red-tailed hawk/Kansas/W22-198/2022	red-tailed hawk
OP950297	4 (HA)	Venezuela	A/Pelecanus occidentalis/Venezuela/Pel3S4/2022	Pelecanus occidentalis
OP950305	4 (HA)	Venezuela	A/Pelecanus occidentalis/Venezuela/Pel4S4/2022	Pelecanus occidentalis

OQ352548	4 (HA)	Chile	A/black skimmer/Chile/C61962/2022	black skimmer
OQ352556	4 (HA)	Chile	A/gray gull/Chile/C61947/2022	gray gull
OQ442195	4 (HA)	USA	A/red-shouldered hawk/North Carolina/W22-121/2022	red-shouldered hawk
OQ455396	4 (HA)	Chile	A/Pelican/CHL/226955-1/2022	Pelican
OQ455401	4 (HA)	Chile	A/Pelican/CHL/226955-3/2022	Pelican
OQ455410	4 (HA)	Chile	A/Pelican/CHL/226618-2/2022	Pelican
OQ455416	4 (HA)	Chile	A/Pelican/CHL/226618-1/2022	Pelican
OQ455422	4 (HA)	Chile	A/Pelican/CHL/226958-1/2022	Pelican
OQ455437	4 (HA)	Chile	A/Gull/CHL/227023-2/2022	Gull
OQ455442	4 (HA)	Chile	A/Gull/CHL/227023-3/2022	Gull
OQ455458	4 (HA)	Chile	A/Pelican/CHL/227087-1/2022	Pelican
OQ455466	4 (HA)	Chile	A/Pelican/CHL/226924-1/2022	Pelican
OQ458189	4 (HA)	Chile	A/Pelican/CHL/227023-1/2022	Pelican
OQ584512	4 (HA)	USA	A/black vulture/Georgia/W22-722B/2022	black vulture
OQ584520	4 (HA)	USA	A/black vulture/Georgia/W22-404A/2022	black vulture
OQ584528	4 (HA)	USA	A/black vulture/Georgia/W22-750B/2022	black vulture
OQ584536	4 (HA)	USA	A/black vulture/South Carolina/W22-689B/2022	black vulture
OQ584567	4 (HA)	USA	A/black vulture/Georgia/W22-723B/2022	black vulture
OQ584598	4 (HA)	USA	A/black vulture/Virginia/W22-662C/2022	black vulture
OQ584630	4 (HA)	USA	A/bald eagle/Kansas/W22-384/2022	bald eagle
OQ584638	4 (HA)	USA	A/black vulture/Virginia/W22-667D/2022	black vulture
OQ584662	4 (HA)	USA	A/black vulture/Georgia/W22-749C/2022	black vulture
OQ584670	4 (HA)	USA	A/black vulture/Georgia/W22-719A/2022	black vulture
OQ584686	4 (HA)	USA	A/black vulture/Georgia/W22-750A/2022	black vulture
OQ584701	4 (HA)	USA	A/black vulture/Virginia/W22-667C/2022	black vulture
OQ584725	4 (HA)	USA	A/black vulture/Georgia/W22-404B/2022	black vulture
OQ584791	4 (HA)	USA	A/black vulture/Georgia/W22-719C/2022	black vulture
OQ632895	4 (HA)	France	A/chicken/France/21328/2021	chicken
OQ683458	4 (HA)	Colombia	A/chicken/Colombia/Bolivar/3500/2022	chicken
OQ683466	4 (HA)	Colombia	A/chicken/Colombia/Choco/3502/2022	chicken
OQ683474	4 (HA)	Colombia	A/chicken/Colombia/Choco/3504/2022	chicken
OQ683482	4 (HA)	Colombia	A/chicken/Colombia/Cordoba/3499/2022	chicken
OQ683490	4 (HA)	Colombia	A/chicken/Colombia/Magdalena/3503/2022	chicken
OQ683498	4 (HA)	Colombia	A/wild duck/Colombia/Choco/3501/2022	wild duck
OQ694806	4 (HA)	USA	A/black vulture/Georgia/W22-1048/2022	black vulture
OQ694846	4 (HA)	USA	A/snow goose/Kansas/W22-1143/2022	snow goose
OQ694854	4 (HA)	USA	A/black vulture/North Carolina/W22-1078/2022	black vulture
OQ694862	4 (HA)	USA	A/black vulture/South Carolina/W22-1112/2022	black vulture
OQ694870	4 (HA)	USA	A/black vulture/South Carolina/W22-1080B/2022	black vulture
OQ694878	4 (HA)	USA	A/snow goose/Louisana/W22-1163/2022	snow goose
OQ694886	4 (HA)	USA	A/black vulture/Georgia/W22-1049/2022	black vulture
OQ694894	4 (HA)	USA	A/black vulture/Georgia/W22-1057B/2022	black vulture
OQ694902	4 (HA)	USA	A/Canada goose/North Carolina/W22-1055/2022	Canada goose
OQ694912	4 (HA)	USA	A/black vulture/Georgia/W22-1057D/2022	black vulture
OQ694920	4 (HA)	USA	A/mallard/North Carolina/W22-1114A/2022	mallard
OQ694928	4 (HA)	USA	A/snow goose/Louisana/W22-1146A/2022	snow goose
OQ694936	4 (HA)	USA	A/black vulture/North Carolina/W22-1051A/2022	black vulture
OQ694944	4 (HA)	USA	A/black vulture/Tennessee/W22-1150B/2022	black vulture
OQ694952	4 (HA)	USA	A/black vulture/Tennessee/W22-1149/2022	black vulture
OQ696052	4 (HA)	USA	A/Ross's goose/Kansas/W22-1154/2022	Ross's goose
OQ696063	4 (HA)	USA	A/red-tailed hawk/Kansas/W22-1155/2022	red-tailed hawk
OQ732940	4 (HA)	USA	A/black vulture/Georgia/W22-969B/2022	black vulture
OQ732956	4 (HA)	USA	A/blue-winged teal/Minnesota/AV22-690/2022	blue-winged teal
OQ732964	4 (HA)	USA	A/red-tailed hawk/Kentucky/W23-143/2022	red-tailed hawk
OQ732972	4 (HA)	USA	A/blue-winged teal/Texas/UGAI22-2966/2022	blue-winged teal
OQ732980	4 (HA)	USA	A/black vulture/Georgia/W22-933C/2022	black vulture
OQ732988	4 (HA)	USA	A/bald eagle/North Carolina/W23-142B/2023	bald eagle
OQ732996	4 (HA)	USA	A/blue-winged teal/Texas/UGAI22-3268/2022	blue-winged teal
OQ733004	4 (HA)	USA	A/blue-winged teal/Minnesota/UGAI22-3620/2022	blue-winged teal
OQ733012	4 (HA)	USA	A/blue-winged teal/Texas/UGAI22-3250/2022	blue-winged teal
OQ733028	4 (HA)	USA	A/blue-winged teal/Texas/UGAI22-3189/2022	blue-winged teal
OQ733036	4 (HA)	USA	A/blue-winged teal/Minnesota/AV22-675/2022	blue-winged teal
OQ733044	4 (HA)	USA	A/black vulture/North Carolina/W22-1079C/2022	black vulture
OQ733052	4 (HA)	USA	A/black vulture/Louisiana/W23-119/2023	black vulture
OQ733060	4 (HA)	USA	A/black vulture/Georgia/W22-972A/2022	black vulture
OQ733068	4 (HA)	USA	A/bald eagle/South Carolina/W23-142A/2023	bald eagle
OQ733076	4 (HA)	USA	A/blue-winged teal/Texas/UGAI22-3226/2022	blue-winged teal
OQ733092	4 (HA)	USA	A/bald eagle/Virginia/W22-101/2023	bald eagle
OQ733100	4 (HA)	USA	A/blue-winged teal/Texas/UGAI22-3190/2022	blue-winged teal
OQ733108	4 (HA)	USA	A/blue-winged teal/Texas/UGAI22-2961/2022	blue-winged teal
OQ733116	4 (HA)	USA	A/black vulture/Georgia/W22-1056A/2022	black vulture
OQ733124	4 (HA)	USA	A/blue-winged teal/Minnesota/UGAI22-3611/2022	blue-winged teal
OQ733140	4 (HA)	USA	A/Canada goose/Virginia/W22-773/2022	Canada goose
OQ733148	4 (HA)	USA	A/blue-winged teal/Minnesota/AV22-680/2022	blue-winged teal
OQ733156	4 (HA)	USA	A/mallard duck/Minnesota/AV22-632/2022	mallard duck
OQ734878	4 (HA)	USA	A/red-tailed hawk/Kansas/W22-1226/2022	red-tailed hawk
OQ734894	4 (HA)	USA	A/American green-winged teal/Texas/UGAI22-3462/2022	American green-winged teal
OQ734902	4 (HA)	USA	A/Cooper's hawk/Kansas/W22-1206/2022	Cooper's hawk
OQ734918	4 (HA)	USA	A/brown pelican/North Carolina/W23-019/2022	brown pelican
OQ734937	4 (HA)	USA	A/herring gull/North Carolina/W1215B/2022	herring gull
OQ734964	4 (HA)	USA	A/black vulture/North Carolina/W22-1213/2022	black vulture
OQ734972	4 (HA)	USA	A/bald eagle/Virginia/W22-1222/2022	bald eagle
OQ737769	4 (HA)	USA	A/great black-backed gull/North Carolina/W22-1215A/2022	great black-backed gull
OQ747758	4 (HA)	Peru	A/Peruvian_pelican/Peru/A074/2022	Peruvian_pelican
OQ747759	4 (HA)	Peru	A/Belcher's_gull/Peru/A102/2022	Belcher's_gull
OQ747760	4 (HA)	Peru	A/Peruvian_pelican/Peru/A106/2022	Peruvian_pelican
OQ747761	4 (HA)	Peru	A/Belcher's_gull/Peru/A267/2022	Belcher's_gull
OQ747762	4 (HA)	Peru	A/American kestrel/Peru/A273/2022	American kestrel
OQ789275	4 (HA)	USA	A/American wigeon/South Carolina/AH0195145/2021	American wigeon
OQ851647	4 (HA)	USA	A/Pekin duck/California/T2202390/2022	Pekin duck
OQ954536	4 (HA)	USA	A/black vulture/Louisiana/W23-166/2023	black vulture
OQ982388	4 (HA)	USA	A/mallard duck/Minnesota/UGAI22-3846/2022	mallard duck
OQ982396	4 (HA)	USA	A/bald eagle/North Carolina/W23-012/2022	bald eagle
OQ982404	4 (HA)	USA	A/blue-winged teal/Louisiana/UGAI22-3889/2022	blue-winged teal
OQ982412	4 (HA)	USA	A/blue-winged teal/Minnesota/UGAI22-3834/2022	blue-winged teal
OQ982420	4 (HA)	USA	A/blue-winged teal/Louisiana/UGAI22-3875/2022	blue-winged teal
OP269982	5 (NP)	USA	A/Vulpes vulpes/USA	Vulpes vulpes
OP377529	5 (NP)	USA	A/common eiders/Maine/W22-481B/2022(H5N1)	Somateria mollissima
OP597634	5 (NP)	Russia	A/pelican/Tumen/932-1/2021(H5N1)	pelican
OQ428780	5 (NP)	USA	A/Anas platyrhynchos/AZ/S1-AZMCP12122022-S5/2022(H5N1)	Anas platyrhynchos
OQ584578	5 (NP)	USA	A/black vulture/South Carolina/W22-623/2022(H5N1)	Coragyps atratus
OQ632844	5 (NP)	France	A/Mule-duck/France/21343/2021(H5N1)	Mule-duck
OQ632850	5 (NP)	France	A/Mule-duck/France/21352/2021(H5N1)	Mule-duck
OQ632851	5 (NP)	France	A/Mule-duck/France/21356/2021(H5N1)	Mule-duck
OQ730466	5 (NP)	USA	A/harbor seal/Maine/MME-22-150Pv-ns/2022(H5N1)	Phoca vitulina
OQ734951	5 (NP)	USA	A/great-tailed grackle/Kansas/W22-1223B/2022(H5N1)	Quiscalus mexicanus
OQ954547	5 (NP)	USA	A/striped skunk/Kansas/W23-175/2023(H5N1)	Mephitis mephitis
OR125145	5 (NP)	Chile	A/Blackish oystercatcher/OHiggins/240628/2023(H5N1)	Haematopus ater

OR125176	5 (NP)	Chile	A/chicken/Nuble/239136/2023(H5N1)	Gallus gallus
OR125352	5 (NP)	Chile	A/Turkey/Araucania/241892-3/2023(H5N1)	Meleagris gallopavo
OR125477	5 (NP)	Chile	A/Humboldt penguin/Tarapaca/238744-2/2023(H5N1)	Spheniscus humboldti
OR125496	5 (NP)	Chile	A/Domestic duck/Araucania/239189-3/2023(H5N1)	Anas platyrhynchos domesticus
OR136565	5 (NP)	USA	A/chicken/OH/OH22-20542-2/2022(H5N1)	Chicken; Gallus gallus domesticus
OR136573	5 (NP)	USA	A/chicken/OH/OH22-21172-2/2022(H5N1)	Chicken; Gallus gallus domesticus
OR136613	5 (NP)	USA	A/goose/OH/OH22-21298/2022(H5N1)	Goose; Anser cygnoides domesticus
OR165065	5 (NP)	USA	A/black vulture/Georgia/W22-406/2022(H5N1)	Coragyps atratus
OP221044	5 (NP)	USA	A/bald eagle/Kansas/W22-197/2022	bald eagle
OP221314	5 (NP)	USA	A/bald eagle/Florida/W22-191/2022	bald eagle
OP221346	5 (NP)	USA	A/American pelican/Kansas/W22-200/2022	American pelican
OP221401	5 (NP)	USA	A/bald eagle/Florida/W22-189/2022	bald eagle
OP377325	5 (NP)	USA	A/snow goose/North Dakota/N22-06/2022	snow goose
OP377367	5 (NP)	USA	A/snow goose/North Dakota/N22-05/2022	snow goose
OP377415	5 (NP)	USA	A/royal tern/Florida/W22-245B/2022	royal tern
OP377521	5 (NP)	USA	A/snow goose/Kansas/W22-260/2022	snow goose
OP377561	5 (NP)	USA	A/black vulture/North Carolina/W22-367C/2022	black vulture
OP377625	5 (NP)	USA	A/bald eagle/North Carolina/W22-140/2022	bald eagle
OP470784	5 (NP)	USA	A/lesser scaup/Georgia/W22-145E/2022	lesser scaup
OP470792	5 (NP)	USA	A/snow goose/Kentucky/W22-092/2022	snow goose
OP950298	5 (NP)	Venezuela	A/Pelecanus occidentalis/Venezuela/Pel3S5/2022	Pelecanus occidentalis
OQ442198	5 (NP)	USA	A/red-shouldered hawk/North Carolina/W22-121/2022	red-shouldered hawk
OQ455443	5 (NP)	Chile	A/Gull/CHL/227023-3/2022	Gull
OQ547313	5 (NP)	Peru	A/Gallus gallus/Peru/AIS0545/2022	Gallus gallus
OQ584539	5 (NP)	USA	A/black vulture/South Carolina/W22-689B/2022	black vulture
OQ584585	5 (NP)	USA	A/black vulture/Virginia/W22-667A/2022	black vulture
OQ584673	5 (NP)	USA	A/black vulture/Georgia/W22-719A/2022	black vulture
OQ632899	5 (NP)	France	A/chicken/France/21328/2021	chicken
OQ683459	5 (NP)	Colombia	A/chicken/Colombia/Bolivar/3500/2022	chicken
OQ683467	5 (NP)	Colombia	A/chicken/Colombia/Choco/3502/2022	chicken
OQ683475	5 (NP)	Colombia	A/chicken/Colombia/Choco/3504/2022	chicken
OQ683491	5 (NP)	Colombia	A/chicken/Colombia/Magdalena/3503/2022	chicken
OQ683499	5 (NP)	Colombia	A/wild duck/Colombia/Choco/3501/2022	wild duck
OQ694849	5 (NP)	USA	A/snow goose/Kansas/W22-1143/2022	snow goose
OQ694865	5 (NP)	USA	A/black vulture/South Carolina/W22-1112/2022	black vulture
OQ694873	5 (NP)	USA	A/black vulture/South Carolina/W22-1080B/2022	black vulture
OQ694881	5 (NP)	USA	A/snow goose/Louisana/W22-1163/2022	snow goose
OQ694905	5 (NP)	USA	A/Canada goose/North Carolina/W22-1055/2022	Canada goose
OQ694923	5 (NP)	USA	A/mallard/North Carolina/W22-1114A/2022	mallard
OQ694931	5 (NP)	USA	A/snow goose/Louisana/W22-1146A/2022	snow goose
OQ694955	5 (NP)	USA	A/black vulture/Tennessee/W22-1149/2022	black vulture
OQ696055	5 (NP)	USA	A/Ross's goose/Kansas/W22-1154/2022	Ross's goose
OQ732975	5 (NP)	USA	A/blue-winged teal/Texas/UGAI22-2966/2022	blue-winged teal
OQ732983	5 (NP)	USA	A/black vulture/Georgia/W22-933C/2022	black vulture
OQ732999	5 (NP)	USA	A/blue-winged teal/Texas/UGAI22-3268/2022	blue-winged teal
OQ733007	5 (NP)	USA	A/blue-winged teal/Minnesota/UGAI22-3620/2022	blue-winged teal
OQ733015	5 (NP)	USA	A/blue-winged teal/Texas/UGAI22-3250/2022	blue-winged teal
OQ733031	5 (NP)	USA	A/blue-winged teal/Texas/UGAI22-3189/2022	blue-winged teal
OQ733063	5 (NP)	USA	A/black vulture/Georgia/W22-972A/2022	black vulture
OQ733071	5 (NP)	USA	A/bald eagle/South Carolina/W23-142A/2023	bald eagle
OQ733135	5 (NP)	USA	A/black vulture/Louisiana/W23-118/2023	black vulture
OQ733151	5 (NP)	USA	A/blue-winged teal/Minnesota/AV22-680/2022	blue-winged teal
OQ734881	5 (NP)	USA	A/red-tailed hawk/Kansas/W22-1226/2022	red-tailed hawk
OQ734897	5 (NP)	USA	A/American green-winged teal/Texas/UGAI22-3462/2022	American green-winged teal
OQ734921	5 (NP)	USA	A/brown pelican/North Carolina/W23-019/2022	brown pelican
OQ734929	5 (NP)	USA	A/double-crested cormorant/North Carolina/W22-1215C/2022	double-crested cormorant
OQ734940	5 (NP)	USA	A/herring gull/North Carolina/W1215B/2022	herring gull
OQ734975	5 (NP)	USA	A/bald eagle/Virginia/W22-1222/2022	bald eagle
OQ851650	5 (NP)	USA	A/Pekin duck/California/T2202390/2022	Pekin duck
OQ982391	5 (NP)	USA	A/mallard duck/Minnesota/UGAI22-3846/2022	mallard duck
OQ982415	5 (NP)	USA	A/blue-winged teal/Minnesota/UGAI22-3834/2022	blue-winged teal
OL636394	6 (NA)	Czech Republic	A/goose/Czech Republic/18520-1/2021(H5N1)	goose
OP269954	6 (NA)	USA	A/Vulpes vulpes/USA	Vulpes vulpes
OP377527	6 (NA)	USA	A/common eiders/Maine/W22-481B/2022(H5N1)	Somateria mollissima
OP597614	6 (NA)	Russia	A/common teal/Chelyabinsk/1379-1/2021(H5N1)	common teal
OP597622	6 (NA)	Russia	A/goose/Chelyabinsk/1341-3/2021(H5N1)	goose
OP597638	6 (NA)	Russia	A/pelican/Tumen/932-1/2021(H5N1)	pelican
OP691326	6 (NA)	Mexico	A/Falco_rusticolus/EdoMex/CPA-19638-22/2022(H5N1)	Falco_rusticolus
OQ428781	6 (NA)	USA	A/Anas platyrhynchos/AZ/S1-AZMCP12122022-S6/2022(H5N1)	Anas platyrhynchos
OQ584498	6 (NA)	USA	A/black vulture/Georgia/W22-723A/2022(H5N1)	Coragyps atratus
OQ584576	6 (NA)	USA	A/black vulture/South Carolina/W22-623/2022(H5N1)	Coragyps atratus
OQ632853	6 (NA)	France	A/Mule-duck/France/21343/2021(H5N1)	Mule-duck
OQ632854	6 (NA)	France	A/Mule-duck/France/21347/2021(H5N1)	Mule-duck
OQ632855	6 (NA)	France	A/Mule-duck/France/21348/2021(H5N1)	Mule-duck
OQ632856	6 (NA)	France	A/Mule-duck/France/21349/2021(H5N1)	Mule-duck
OQ632857	6 (NA)	France	A/Mule-duck/France/21356/2021(H5N1)	Mule-duck
OQ632861	6 (NA)	France	A/Mule-duck/France/22027/2022(H5N1)	Mule-duck
OQ632862	6 (NA)	France	A/Mule-duck/France/22030/2022(H5N1)	Mule-duck
OQ632864	6 (NA)	France	A/Pekin-duck/France/22077/2022(H5N1)	Pekin-duck
OQ632865	6 (NA)	France	A/Mule-duck/France/22083/2022(H5N1)	Mule-duck
OQ632866	6 (NA)	France	A/Turkey/France/22084/2022(H5N1)	turkey
OQ632867	6 (NA)	France	A/Mule-duck/France/21350/2021(H5N1)	Mule-duck
OQ733085	6 (NA)	USA	A/striped skunk/Kansas/W23-094/2023(H5N1)	Mephitis mephitis
OQ734949	6 (NA)	USA	A/great-tailed grackle/Kansas/W22-1223B/2022(H5N1)	Quiscalus mexicanus
OQ734957	6 (NA)	USA	A/great-tailed grackle/Kansas/W22-1223A/2022(H5N1)	Quiscalus mexicanus
OQ954545	6 (NA)	USA	A/striped skunk/Kansas/W23-175/2023(H5N1)	Mephitis mephitis
OR125149	6 (NA)	Chile	A/Whimbrel/Valparaiso/239946/2023(H5N1)	Numenius phaeopus
OR125157	6 (NA)	Chile	A/South American tern/Maule/238507/2023(H5N1)	Sterna hirundinacea
OR125203	6 (NA)	Chile	A/chicken/Nuble/240155/2023(H5N1)	Gallus gallus
OR125363	6 (NA)	Chile	A/Chiloe wigeon/OHiggins/240893-2/2023(H5N1)	Mareca sibilatrix
OR136526	6 (NA)	USA	A/Silver Pheasant/OH/OH22-20552/2022(H5N1)	Silver Pheasant; Lophura nycthemera
OR136542	6 (NA)	USA	A/Turkey/OH/OH22-21172-1/2022(H5N1)	Turkey; Meleagris gallopavo domesticus
OR136558	6 (NA)	USA	A/chicken/OH/OH22-20542-1/2022(H5N1)	Chicken; Gallus gallus domesticus
OR136582	6 (NA)	USA	A/chicken/OH/OH22-26275-1/2022(H5N1)	Chicken; Gallus gallus domesticus
OP221042	6 (NA)	USA	A/bald eagle/Kansas/W22-197/2022	bald eagle
OP221336	6 (NA)	USA	A/bald eagle/Georgia/W22-202/2022	bald eagle
OP377339	6 (NA)	USA	A/black vulture/Virginia/W22-499C/2022	black vulture
OP377413	6 (NA)	USA	A/royal tern/Florida/W22-245B/2022	royal tern
OP377503	6 (NA)	USA	A/common tern/Maine/W22-480A/2022	common tern
OP377535	6 (NA)	USA	A/bald eagle/Florida/W22-153A/2022	bald eagle
OP377559	6 (NA)	USA	A/black vulture/North Carolina/W22-367C/2022	black vulture
OP950299	6 (NA)	Venezuela	A/Pelecanus occidentalis/Venezuela/Pel3S6/2022	Pelecanus occidentalis
OP950307	6 (NA)	Venezuela	A/Pelecanus occidentalis/Venezuela/Pel4S6/2022	Pelecanus occidentalis
OQ352558	6 (NA)	Chile	A/gray gull/Chile/C61947/2022	gray gull
OQ584537	6 (NA)	USA	A/black vulture/South Carolina/W22-689B/2022	black vulture
OQ584545	6 (NA)	USA	A/black vulture/Georgia/W22-675B/2022	black vulture
OQ632900	6 (NA)	France	A/chicken/France/21328/2021	chicken
OQ683468	6 (NA)	Colombia	A/chicken/Colombia/Choco/3502/2022	chicken

OQ683476	6 (NA)	Colombia	A/chicken/Colombia/Choco/3504/2022	chicken
OQ683500	6 (NA)	Colombia	A/wild duck/Colombia/Choco/3501/2022	wild duck
OQ694847	6 (NA)	USA	A/snow goose/Kansas/W22-1143/2022	snow goose
OQ694863	6 (NA)	USA	A/black vulture/South Carolina/W22-1112/2022	black vulture
OQ694879	6 (NA)	USA	A/snow goose/Louisana/W22-1163/2022	snow goose
OQ694903	6 (NA)	USA	A/Canada goose/North Carolina/W22-1055/2022	Canada goose
OQ694921	6 (NA)	USA	A/mallard/North Carolina/W22-1114A/2022	mallard
OQ694929	6 (NA)	USA	A/snow goose/Louisana/W22-1146A/2022	snow goose
OQ694945	6 (NA)	USA	A/black vulture/Tennessee/W22-1150B/2022	black vulture
OQ732957	6 (NA)	USA	A/blue-winged teal/Minnesota/AV22-690/2022	blue-winged teal
OQ732973	6 (NA)	USA	A/blue-winged teal/Texas/UGAI22-2966/2022	blue-winged teal
OQ732981	6 (NA)	USA	A/black vulture/Georgia/W22-933C/2022	black vulture
OQ732997	6 (NA)	USA	A/blue-winged teal/Texas/UGAI22-3268/2022	blue-winged teal
OQ733005	6 (NA)	USA	A/blue-winged teal/Minnesota/UGAI22-3620/2022	blue-winged teal
OQ733013	6 (NA)	USA	A/blue-winged teal/Texas/UGAI22-3250/2022	blue-winged teal
OQ733053	6 (NA)	USA	A/black vulture/Louisiana/W23-119/2023	black vulture
OQ733069	6 (NA)	USA	A/bald eagle/South Carolina/W23-142A/2023	bald eagle
OQ733125	6 (NA)	USA	A/blue-winged teal/Minnesota/UGAI22-3611/2022	blue-winged teal
OQ733149	6 (NA)	USA	A/blue-winged teal/Minnesota/AV22-680/2022	blue-winged teal
OQ734895	6 (NA)	USA	A/American green-winged teal/Texas/UGAI22-3462/2022	American green-winged teal
OQ734919	6 (NA)	USA	A/brown pelican/North Carolina/W23-019/2022	brown pelican
OQ734927	6 (NA)	USA	A/double-crested cormorant/North Carolina/W22-1215C/2022	double-crested cormorant
OQ734938	6 (NA)	USA	A/herring gull/North Carolina/W1215B/2022	herring gull
OQ734965	6 (NA)	USA	A/black vulture/North Carolina/W22-1213/2022	black vulture
OQ734973	6 (NA)	USA	A/bald eagle/Virginia/W22-1222/2022	bald eagle
OQ737770	6 (NA)	USA	A/great black-backed gull/North Carolina/W22-1215A/2022	great black-backed gull
OQ982405	6 (NA)	USA	A/blue-winged teal/Louisiana/UGAI22-3889/2022	blue-winged teal
ON759333	7 (MP)	USA	A/Colorado/18/2022(H5N1)	Homo sapiens
OP269958	7 (MP)	USA	A/Vulpes vulpes/USA	Vulpes vulpes
OP270009	7 (MP)	USA	A/Vulpes vulpes/USA	Vulpes vulpes
OP377463	7 (MP)	USA	A/bald eagle/Florida/W22-153B/2022(H5N1)	Haliaeetus leucocephalus
OP377528	7 (MP)	USA	A/common eiders/Maine/W22-481B/2022(H5N1)	Somateria mollissima
OP597613	7 (MP)	Russia	A/common teal/Chelyabinsk/1379-1/2021(H5N1)	common teal
OP597629	7 (MP)	Russia	A/pelican/Tumen/1032-1/2021(H5N1)	pelican
OP597637	7 (MP)	Russia	A/pelican/Tumen/932-1/2021(H5N1)	pelican
OP691327	7 (MP)	Mexico	A/Falco_rusticolus/EdoMex/CPA-19638-22/2022(H5N1)	Falco_rusticolus
OP698131	7 (MP)	USA	A/bottlenose dolphin/Florida/UFTt2203/2022(H5N1)	Tursiops truncatus
OQ428782	7 (MP)	USA	A/Anas platyrhynchos/AZ/S1-AZMCP12122022-S7/2022(H5N1)	Anas platyrhynchos
OQ584562	7 (MP)	USA	A/bald eagle/North Carolina/W22-729/2022(H5N1)	Haliaeetus leucocephalus
OQ584577	7 (MP)	USA	A/black vulture/South Carolina/W22-623/2022(H5N1)	Coragyps atratus
OQ595419	7 (MP)	USA	A/black vulture/Georgia/W22-723A/2022(H5N1)	Coragyps atratus
OQ632868	7 (MP)	France	A/Mule-duck/France/21343/2021(H5N1)	Mule-duck
OQ632869	7 (MP)	France	A/Mule-duck/France/21347/2021(H5N1)	Mule-duck
OQ632874	7 (MP)	France	A/Mule-duck/France/21352/2021(H5N1)	Mule-duck
OQ730458	7 (MP)	USA	A/harbor seal/Maine/MME-22-147Pv-L/2022(H5N1)	Phoca vitulina
OQ730497	7 (MP)	USA	A/harbor seal/Maine/MME-22-185Pv-ns/2022(H5N1)	Phoca vitulina
OQ733086	7 (MP)	USA	A/striped skunk/Kansas/W23-094/2023(H5N1)	Mephitis mephitis
OQ734912	7 (MP)	USA	A/great-tailed grackle/Kansas/W22-1223C/2022(H5N1)	Quiscalus mexicanus
OQ734950	7 (MP)	USA	A/great-tailed grackle/Kansas/W22-1223B/2022(H5N1)	Quiscalus mexicanus
OQ734958	7 (MP)	USA	A/great-tailed grackle/Kansas/W22-1223A/2022(H5N1)	Quiscalus mexicanus
OQ843968	7 (MP)	USA	A/environment/Wright County/Wright-A/2022(H5N1)	NA
OQ954546	7 (MP)	USA	A/striped skunk/Kansas/W23-175/2023(H5N1)	Mephitis mephitis
OR125150	7 (MP)	Chile	A/Whimbrel/Valparaiso/239946/2023(H5N1)	Numenius phaeopus
OR125158	7 (MP)	Chile	A/South American tern/Maule/238507/2023(H5N1)	Sterna hirundinacea
OR125170	7 (MP)	Chile	A/Humboldt penguin/Coquimbo/239590/2023(H5N1)	Spheniscus humboldti
OR125191	7 (MP)	Chile	A/Sanderling/Arica y Parinacota/240265/2023(H5N1)	Calidris alba
OR125196	7 (MP)	Chile	A/Black-crowned night-heron/Antofagasta/228705-2/2022(H5N1)	Nycticorax nycticorax
OR125211	7 (MP)	Chile	A/Black Skimmer/Maule/240379/2023(H5N1)	Rynchops niger
OR125235	7 (MP)	Chile	A/Pelican/Antofagasta/228246-2/2022(H5N1)	Pelecanus sp.
OR125315	7 (MP)	Chile	A/Pelican/Atacama/229424-2/2022(H5N1)	Pelecanus sp.
OR125330	7 (MP)	Chile	A/chicken/OHiggins/241252-1/2023(H5N1)	Gallus gallus
OR125336	7 (MP)	Chile	A/goose/Araucania/239189-1/2023(H5N1)	Anser sp.
OR125353	7 (MP)	Chile	A/Turkey/Araucania/241892-3/2023(H5N1)	Meleagris gallopavo
OR125364	7 (MP)	Chile	A/Chiloe wigeon/OHiggins/240893-2/2023(H5N1)	Mareca sibilatrix
OR125386	7 (MP)	Chile	A/chicken/Nuble/241681-1/2023(H5N1)	Gallus gallus
OR125417	7 (MP)	Chile	A/chicken/Nuble/241557-1/2023(H5N1)	Gallus gallus
OR125448	7 (MP)	Chile	A/chicken/Araucania/241892-2/2023(H5N1)	Gallus gallus
OR125491	7 (MP)	Chile	A/chicken/Araucania/240481-1/2023(H5N1)	Gallus gallus
OR136519	7 (MP)	USA	A/Bald Eagle/OH/OH22-8477/2023(H5N1)	Bald Eagle; Haliaeetus leucocephalus
OR136527	7 (MP)	USA	A/Silver Pheasant/OH/OH22-20552/2022(H5N1)	Silver Pheasant; Lophura nycthemera
OR136535	7 (MP)	USA	A/Swan/OH/OH22-22368-2/2022(H5N1)	Swan; Cygnus sp.
OR136543	7 (MP)	USA	A/Turkey/OH/OH22-21172-1/2022(H5N1)	Turkey; Meleagris gallopavo domesticus
OR136559	7 (MP)	USA	A/chicken/OH/OH22-20542-1/2022(H5N1)	Chicken; Gallus gallus domesticus
OR136567	7 (MP)	USA	A/chicken/OH/OH22-20542-2/2022(H5N1)	Chicken; Gallus gallus domesticus
OR136607	7 (MP)	USA	A/chicken/OH/OH22-7075/2022(H5N1)	Chicken; Gallus gallus domesticus
OR136610	7 (MP)	USA	A/goose/OH/OH22-21298/2022(H5N1)	Goose; Anser cygnoides domesticus
OR165048	7 (MP)	USA	A/common eider/Massachusetts/W22-438A/2022(H5N1)	Somateria mollissima
OR165064	7 (MP)	USA	A/black vulture/Georgia/W22-406/2022(H5N1)	Coragyps atratus
OR165072	7 (MP)	USA	A/black vulture/Georgia/W22-487/2022(H5N1)	Coragyps atratus
OP221043	7 (MP)	USA	A/bald eagle/Kansas/W22-197/2022	bald eagle
OP221287	7 (MP)	USA	A/snow goose/Kansas/W22-199D/2022	snow goose
OP221303	7 (MP)	USA	A/bald eagle/Georgia/W22-194B/2022	bald eagle
OP221352	7 (MP)	USA	A/snow goose/Kansas/W22-199A/2022	snow goose
OP221368	7 (MP)	USA	A/bald eagle/South Carolina/W22-205/2022	bald eagle
OP221376	7 (MP)	USA	A/snow goose/Kansas/W22-199E/2022	snow goose
OP377324	7 (MP)	USA	A/snow goose/North Dakota/N22-06/2022	snow goose
OP377332	7 (MP)	USA	A/snow goose/North Dakota/N22-04/2022	snow goose
OP377340	7 (MP)	USA	A/black vulture/Virginia/W22-499C/2022	black vulture
OP377398	7 (MP)	USA	A/muscovy duck/Florida/W22-306/2022	muscovy duck
OP377422	7 (MP)	USA	A/gull/Florida/W22-162/2022	gull
OP377447	7 (MP)	USA	A/bald eagle/North Carolina/W22-229/2022	bald eagle
OP377504	7 (MP)	USA	A/common tern/Maine/W22-480A/2022	common tern
OP377552	7 (MP)	USA	A/common tern/Maine/W22-480B/2022	common tern
OP377584	7 (MP)	USA	A/lesser snow goose/North Dakota/ND-10/2022	lesser snow goose
OP377592	7 (MP)	USA	A/snow goose/Kansas/W22-174B/2022	snow goose
OP377608	7 (MP)	USA	A/brown pelican/North Carolina/W22-164/2022	brown pelican
OP377624	7 (MP)	USA	A/bald eagle/North Carolina/W22-140/2022	bald eagle
OP377640	7 (MP)	USA	A/great horned owl/Flordia/W22-163C/2022	great horned owl
OP470718	7 (MP)	USA	A/lesser scaup/Georgia/W22-145C/2022	lesser scaup
OP470751	7 (MP)	USA	A/lesser scaup/Georgia/W22-145B/2022	lesser scaup
OP470767	7 (MP)	USA	A/lesser scaup/Georgia/W22-143/2022	lesser scaup
OP470783	7 (MP)	USA	A/lesser scaup/Georgia/W22-145E/2022	lesser scaup
OP470791	7 (MP)	USA	A/snow goose/Kentucky/W22-092/2022	snow goose
OP470814	7 (MP)	USA	A/lesser scaup/Florida/W22-129A/2022	lesser scaup
OP950300	7 (MP)	Venezuela	A/Pelecanus occidentalis/Venezuela/Pel3S7/2022	Pelecanus occidentalis
OP950308	7 (MP)	Venezuela	A/Pelecanus occidentalis/Venezuela/Pel4S7/2022	Pelecanus occidentalis
OQ352543	7 (MP)	Chile	A/Peruvian pelican/Chile/C61740/2022	Peruvian pelican
OQ352551	7 (MP)	Chile	A/black skimmer/Chile/C61962/2022	black skimmer

OQ455408	7 (MP)	Chile	A/Pelican/CHL/227023-1/2022	Pelican
OQ455412	7 (MP)	Chile	A/Pelican/CHL/226618-2/2022	Pelican
OQ455427	7 (MP)	Chile	A/Pelican/CHL/226924-1/2022	Pelican
OQ455433	7 (MP)	Chile	A/Pelican/CHL/226618-1/2022	Pelican
OQ455461	7 (MP)	Chile	A/Pelican/CHL/227087-1/2022	Pelican
OQ547330	7 (MP)	Peru	A/Nannopterum brasilianus/Peru/AISA0451/2022	Nannopterum brasilianus
OQ547338	7 (MP)	Peru	A/Pelecanus thagus/Peru/AIS0541/2022	Pelecanus thagus
OQ547346	7 (MP)	Peru	A/Gallus gallus/Peru/AIS0539/2022	Gallus gallus
OQ547362	7 (MP)	Peru	A/Gallus gallus/Peru/AIS0542/2022	Gallus gallus
OQ547394	7 (MP)	Peru	A/Gallus gallus/Peru/AIS0548/2022	Gallus gallus
OQ547410	7 (MP)	Peru	A/Gallus gallus/Peru/AIS0550/2022	Gallus gallus
OQ547442	7 (MP)	Peru	A/Pelecanus thagus/Peru/AIS0538/2022	Pelecanus thagus
OQ547450	7 (MP)	Peru	A/Pelecanus thagus/Peru/AISA0464/2022	Pelecanus thagus
OQ565631	7 (MP)	Peru	A/Pelecanus/Peru/VFAR-140/2022	Pelecanus
OQ584522	7 (MP)	USA	A/black vulture/Georgia/W22-404A/2022	black vulture
OQ584538	7 (MP)	USA	A/black vulture/South Carolina/W22-689B/2022	black vulture
OQ584584	7 (MP)	USA	A/black vulture/Virginia/W22-667A/2022	black vulture
OQ584680	7 (MP)	USA	A/black vulture/Georgia/W22-749B/2022	black vulture
OQ584727	7 (MP)	USA	A/black vulture/Georgia/W22-404B/2022	black vulture
OQ584793	7 (MP)	USA	A/black vulture/Georgia/W22-719C/2022	black vulture
OQ632901	7 (MP)	France	A/chicken/France/21328/2021	chicken
OQ683461	7 (MP)	Colombia	A/chicken/Colombia/Bolivar/3500/2022	chicken
OQ683469	7 (MP)	Colombia	A/chicken/Colombia/Choco/3502/2022	chicken
OQ683477	7 (MP)	Colombia	A/chicken/Colombia/Choco/3504/2022	chicken
OQ683485	7 (MP)	Colombia	A/chicken/Colombia/Cordoba/3499/2022	chicken
OQ683493	7 (MP)	Colombia	A/chicken/Colombia/Magdalena/3503/2022	chicken
OQ683501	7 (MP)	Colombia	A/wild duck/Colombia/Choco/3501/2022	wild duck
OQ694848	7 (MP)	USA	A/snow goose/Kansas/W22-1143/2022	snow goose
OQ694856	7 (MP)	USA	A/black vulture/North Carolina/W22-1078/2022	black vulture
OQ694864	7 (MP)	USA	A/black vulture/South Carolina/W22-1112/2022	black vulture
OQ694872	7 (MP)	USA	A/black vulture/South Carolina/W22-1080B/2022	black vulture
OQ694880	7 (MP)	USA	A/snow goose/Louisana/W22-1163/2022	snow goose
OQ694888	7 (MP)	USA	A/black vulture/Georgia/W22-1049/2022	black vulture
OQ694904	7 (MP)	USA	A/Canada goose/North Carolina/W22-1055/2022	Canada goose
OQ694922	7 (MP)	USA	A/mallard/North Carolina/W22-1114A/2022	mallard
OQ694930	7 (MP)	USA	A/snow goose/Louisana/W22-1146A/2022	snow goose
OQ694938	7 (MP)	USA	A/black vulture/North Carolina/W22-1051A/2022	black vulture
OQ694954	7 (MP)	USA	A/black vulture/Tennessee/W22-1149/2022	black vulture
OQ696054	7 (MP)	USA	A/Ross's goose/Kansas/W22-1154/2022	Ross's goose
OQ732942	7 (MP)	USA	A/black vulture/Georgia/W22-969B/2022	black vulture
OQ732958	7 (MP)	USA	A/blue-winged teal/Minnesota/AV22-690/2022	blue-winged teal
OQ732966	7 (MP)	USA	A/red-tailed hawk/Kentucky/W23-143/2022	red-tailed hawk
OQ732974	7 (MP)	USA	A/blue-winged teal/Texas/UGAI22-2966/2022	blue-winged teal
OQ732982	7 (MP)	USA	A/black vulture/Georgia/W22-933C/2022	black vulture
OQ732998	7 (MP)	USA	A/blue-winged teal/Texas/UGAI22-3268/2022	blue-winged teal
OQ733006	7 (MP)	USA	A/blue-winged teal/Minnesota/UGAI22-3620/2022	blue-winged teal
OQ733014	7 (MP)	USA	A/blue-winged teal/Texas/UGAI22-3250/2022	blue-winged teal
OQ733022	7 (MP)	USA	A/bald eagle/North Carolina/W23-142A/2023	bald eagle
OQ733030	7 (MP)	USA	A/blue-winged teal/Texas/UGAI22-3189/2022	blue-winged teal
OQ733062	7 (MP)	USA	A/black vulture/Georgia/W22-972A/2022	black vulture
OQ733070	7 (MP)	USA	A/bald eagle/South Carolina/W23-142A/2023	bald eagle
OQ733078	7 (MP)	USA	A/blue-winged teal/Texas/UGAI22-3226/2022	blue-winged teal
OQ733094	7 (MP)	USA	A/bald eagle/Virginia/W22-101/2023	bald eagle
OQ733110	7 (MP)	USA	A/blue-winged teal/Texas/UGAI22-2961/2022	blue-winged teal
OQ733118	7 (MP)	USA	A/black vulture/Georgia/W22-1056A/2022	black vulture
OQ733126	7 (MP)	USA	A/blue-winged teal/Minnesota/UGAI22-3611/2022	blue-winged teal
OQ733134	7 (MP)	USA	A/black vulture/Louisiana/W23-118/2023	black vulture
OQ733150	7 (MP)	USA	A/blue-winged teal/Minnesota/AV22-680/2022	blue-winged teal
OQ734880	7 (MP)	USA	A/red-tailed hawk/Kansas/W22-1226/2022	red-tailed hawk
OQ734896	7 (MP)	USA	A/American green-winged teal/Texas/UGAI22-3462/2022	American green-winged teal
OQ734904	7 (MP)	USA	A/Cooper's hawk/Kansas/W22-1206/2022	Cooper's hawk
OQ734920	7 (MP)	USA	A/brown pelican/North Carolina/W23-019/2022	brown pelican
OQ734928	7 (MP)	USA	A/double-crested cormorant/North Carolina/W22-1215C/2022	double-crested cormorant
OQ734939	7 (MP)	USA	A/herring gull/North Carolina/W1215B/2022	herring gull
OQ734966	7 (MP)	USA	A/black vulture/North Carolina/W22-1213/2022	black vulture
OQ734974	7 (MP)	USA	A/bald eagle/Virginia/W22-1222/2022	bald eagle
OQ747896	7 (MP)	Peru	A/Belcher's_gull/Peru/A102/2022	Belcher's_gull
OQ747898	7 (MP)	Peru	A/Belcher's_gull/Peru/A267/2022	Belcher's_gull
OQ789278	7 (MP)	USA	A/American wigeon/South Carolina/AH0195145/2021	American wigeon
OQ851648	7 (MP)	USA	A/Pekin duck/California/T2202390/2022	Pekin duck
OQ982390	7 (MP)	USA	A/mallard duck/Minnesota/UGAI22-3846/2022	mallard duck
OQ982398	7 (MP)	USA	A/bald eagle/North Carolina/W23-012/2022	bald eagle
OQ982406	7 (MP)	USA	A/blue-winged teal/Louisiana/UGAI22-3889/2022	blue-winged teal
OQ982414	7 (MP)	USA	A/blue-winged teal/Minnesota/UGAI22-3834/2022	blue-winged teal
OQ982422	7 (MP)	USA	A/blue-winged teal/Louisiana/UGAI22-3875/2022	blue-winged teal
OP377465	8 (NS)	USA	A/bald eagle/Florida/W22-153B/2022(H5N1)	Haliaeetus leucocephalus
OP470801	8 (NS)	USA	A/bald eagle/Florida/W22-142/2022(H5N1)	Haliaeetus leucocephalus
OP499864	8 (NS)	USA	A/Lesser scaup/MD/-LC-EESC-024/2022(H5N1)	Aythya affinis
OQ428783	8 (NS)	USA	A/Anas platyrhynchos/AZ/S1-AZMCP12122022-S8/2022(H5N1)	Anas platyrhynchos
OQ584500	8 (NS)	USA	A/black vulture/Georgia/W22-723A/2022(H5N1)	Coragyps atratus
OQ584564	8 (NS)	USA	A/bald eagle/North Carolina/W22-729/2022(H5N1)	Haliaeetus leucocephalus
OQ584579	8 (NS)	USA	A/black vulture/South Carolina/W22-623/2022(H5N1)	Coragyps atratus
OQ584697	8 (NS)	USA	A/black vulture/Georgia/W22-619B/2022(H5N1)	Coragyps atratus
OP221045	8 (NS)	USA	A/bald eagle/Kansas/W22-197/2022	bald eagle
OP221289	8 (NS)	USA	A/snow goose/Kansas/W22-199D/2022	snow goose
OP221297	8 (NS)	USA	A/snow goose/Kansas/W22-199F/2022	snow goose
OP221305	8 (NS)	USA	A/bald eagle/Georgia/W22-194B/2022	bald eagle
OP221315	8 (NS)	USA	A/bald eagle/Florida/W22-191/2022	bald eagle
OP221331	8 (NS)	USA	A/bald eagle/Florida/W22-195/2022	bald eagle
OP221339	8 (NS)	USA	A/bald eagle/Georgia/W22-202/2022	bald eagle
OP221347	8 (NS)	USA	A/American pelican/Kansas/W22-200/2022	American pelican
OP221354	8 (NS)	USA	A/snow goose/Kansas/W22-199A/2022	snow goose
OP221362	8 (NS)	USA	A/snow goose/Kansas/W22-199B/2022	snow goose
OP221370	8 (NS)	USA	A/bald eagle/South Carolina/W22-205/2022	bald eagle
OP221378	8 (NS)	USA	A/snow goose/Kansas/W22-199E/2022	snow goose
OP221386	8 (NS)	USA	A/bald eagle/Georgia/W22-194A/2022	bald eagle
OP221394	8 (NS)	USA	A/snow goose/Kansas/W22-199C/2022	snow goose
OP221402	8 (NS)	USA	A/bald eagle/Florida/W22-189/2022	bald eagle
OP377326	8 (NS)	USA	A/snow goose/North Dakota/N22-06/2022	snow goose
OP377334	8 (NS)	USA	A/snow goose/North Dakota/N22-04/2022	snow goose
OP377342	8 (NS)	USA	A/black vulture/Virginia/W22-499C/2022	black vulture
OP377368	8 (NS)	USA	A/snow goose/North Dakota/N22-05/2022	snow goose
OP377376	8 (NS)	USA	A/ring-billed gull/Florida/W22-169/2022	ring-billed gull
OP377384	8 (NS)	USA	A/bald eagle/Virginia/W22-306/2022	bald eagle
OP377392	8 (NS)	USA	A/black vulture/Virginia/W22-499A/2022	black vulture
OP377400	8 (NS)	USA	A/muscovy duck/Florida/W22-306/2022	muscovy duck
OP377408	8 (NS)	USA	A/bald eagle/North Carolina/W22-186/2022	bald eagle
OP377416	8 (NS)	USA	A/royal tern/Florida/W22-245B/2022	royal tern

OP377424	8 (NS)	USA	A/gull/Florida/W22-162/2022	gull
OP377432	8 (NS)	USA	A/snow goose/Kansas/W22-177B/2022	snow goose
OP377440	8 (NS)	USA	A/bald eagle/Florida/W22-134-OP/2022	bald eagle
OP377449	8 (NS)	USA	A/bald eagle/North Carolina/W22-229/2022	bald eagle
OP377457	8 (NS)	USA	A/black vulture/Florida/W22-168/2022	black vulture
OP377490	8 (NS)	USA	A/black vulture/Florida/W22-161/2022	black vulture
OP377498	8 (NS)	USA	A/black vulture/Virginia/W22-499B/2022	black vulture
OP377506	8 (NS)	USA	A/common tern/Maine/W22-480A/2022	common tern
OP377514	8 (NS)	USA	A/great horned owl/Flordia/W22-163A/2022	great horned owl
OP377522	8 (NS)	USA	A/snow goose/Kansas/W22-260/2022	snow goose
OP377538	8 (NS)	USA	A/bald eagle/Florida/W22-153A/2022	bald eagle
OP377546	8 (NS)	USA	A/great blue heron/Florida/W22-160/2022	great blue heron
OP377554	8 (NS)	USA	A/common tern/Maine/W22-480B/2022	common tern
OP377562	8 (NS)	USA	A/black vulture/North Carolina/W22-367C/2022	black vulture
OP377570	8 (NS)	USA	A/Ross's goose/North Dakota/N22-08/2022	Ross's goose
OP377578	8 (NS)	USA	A/black vulture/Florida/W22-167/2022	black vulture
OP377586	8 (NS)	USA	A/lesser snow goose/North Dakota/ND-10/2022	lesser snow goose
OP377594	8 (NS)	USA	A/snow goose/Kansas/W22-174B/2022	snow goose
OP377610	8 (NS)	USA	A/brown pelican/North Carolina/W22-164/2022	brown pelican
OP377618	8 (NS)	USA	A/royal tern/Florida/W22-245A/2022	royal tern
OP377626	8 (NS)	USA	A/bald eagle/North Carolina/W22-140/2022	bald eagle
OP377634	8 (NS)	USA	A/hooded merganser/Florida/W22-154/2022	hooded merganser
OP377642	8 (NS)	USA	A/great horned owl/Flordia/W22-163C/2022	great horned owl
OP377650	8 (NS)	USA	A/bald eagle/Kansas/W22-185/2022	bald eagle
OP470720	8 (NS)	USA	A/lesser scaup/Georgia/W22-145C/2022	lesser scaup
OP470728	8 (NS)	USA	A/bald eagle/FL/W22-114/2022	bald eagle
OP470737	8 (NS)	USA	A/bald eagle/Florida/W22-134-CL/2022	bald eagle
OP470745	8 (NS)	USA	A/bald eagle/FL/W22-114/2022	bald eagle
OP470753	8 (NS)	USA	A/lesser scaup/Georgia/W22-145B/2022	lesser scaup
OP470761	8 (NS)	USA	A/lesser scaup/Georgia/W22-145D/2022	lesser scaup
OP470769	8 (NS)	USA	A/lesser scaup/Georgia/W22-143/2022	lesser scaup
OP470777	8 (NS)	USA	A/lesser scaup/Georgia/W22-145A/2022	lesser scaup
OP470785	8 (NS)	USA	A/lesser scaup/Georgia/W22-145E/2022	lesser scaup
OP470793	8 (NS)	USA	A/snow goose/Kentucky/W22-092/2022	snow goose
OP470808	8 (NS)	USA	A/bald eagle/FL/W22-114/2022	bald eagle
OP470816	8 (NS)	USA	A/lesser scaup/Florida/W22-129A/2022	lesser scaup
OP499870	8 (NS)	USA	A/red-tailed hawk/Kansas/W22-198/2022	red-tailed hawk
OP950301	8 (NS)	Venezuela	A/Pelecanus occidentalis/Venezuela/Pel3S8/2022	Pelecanus occidentalis
OP950309	8 (NS)	Venezuela	A/Pelecanus occidentalis/Venezuela/Pel4S8/2022	Pelecanus occidentalis
OQ352538	8 (NS)	Chile	A/gray gull/Chile/C61947/2022	gray gull
OQ352544	8 (NS)	Chile	A/Peruvian pelican/Chile/C61740/2022	Peruvian pelican
OQ352552	8 (NS)	Chile	A/black skimmer/Chile/C61962/2022	black skimmer
OQ442199	8 (NS)	USA	A/red-shouldered hawk/North Carolina/W22-121/2022	red-shouldered hawk
OQ455400	8 (NS)	Chile	A/Pelican/CHL/226955-1/2022	Pelican
OQ455404	8 (NS)	Chile	A/Gull/CHL/227023-3/2022	Gull
OQ455409	8 (NS)	Chile	A/Pelican/CHL/227023-1/2022	Pelican
OQ455413	8 (NS)	Chile	A/Pelican/CHL/226618-2/2022	Pelican
OQ455419	8 (NS)	Chile	A/Pelican/CHL/226618-1/2022	Pelican
OQ455425	8 (NS)	Chile	A/Pelican/CHL/226958-1/2022	Pelican
OQ455428	8 (NS)	Chile	A/Pelican/CHL/226924-1/2022	Pelican
OQ455431	8 (NS)	Chile	A/Gull/CHL/227023-2/2022	Gull
OQ455454	8 (NS)	Chile	A/Pelican/CHL/226955-3/2022	Pelican
OQ455462	8 (NS)	Chile	A/Pelican/CHL/227087-1/2022	Pelican
OQ584508	8 (NS)	USA	A/black vulture/Georgia/W22-769/2022	black vulture
OQ584516	8 (NS)	USA	A/black vulture/Georgia/W22-722B/2022	black vulture
OQ584524	8 (NS)	USA	A/black vulture/Georgia/W22-404A/2022	black vulture
OQ584532	8 (NS)	USA	A/black vulture/Georgia/W22-750B/2022	black vulture
OQ584540	8 (NS)	USA	A/black vulture/South Carolina/W22-689B/2022	black vulture
OQ584548	8 (NS)	USA	A/black vulture/Georgia/W22-675B/2022	black vulture
OQ584556	8 (NS)	USA	A/black vulture/Georgia/W22-619A/2022	black vulture
OQ584571	8 (NS)	USA	A/black vulture/Georgia/W22-723B/2022	black vulture
OQ584586	8 (NS)	USA	A/black vulture/Virginia/W22-667A/2022	black vulture
OQ584594	8 (NS)	USA	A/black vulture/Georgia/W22-736A/2022	black vulture
OQ584602	8 (NS)	USA	A/black vulture/Virginia/W22-662C/2022	black vulture
OQ584610	8 (NS)	USA	A/black vulture/Georgia/W22-722C/2022	black vulture
OQ584618	8 (NS)	USA	A/black vulture/Georgia/W22-736B/2022	black vulture
OQ584626	8 (NS)	USA	A/black vulture/Georgia/W22-674/2022	black vulture
OQ584634	8 (NS)	USA	A/bald eagle/Kansas/W22-384/2022	bald eagle
OQ584642	8 (NS)	USA	A/black vulture/Virginia/W22-667D/2022	black vulture
OQ584650	8 (NS)	USA	A/black vulture/Georgia/W22-675C/2022	black vulture
OQ584658	8 (NS)	USA	A/black vulture/Virginia/W22-667B/2022	black vulture
OQ584666	8 (NS)	USA	A/black vulture/Georgia/W22-749C/2022	black vulture
OQ584674	8 (NS)	USA	A/black vulture/Georgia/W22-719A/2022	black vulture
OQ584682	8 (NS)	USA	A/black vulture/Georgia/W22-749B/2022	black vulture
OQ584690	8 (NS)	USA	A/black vulture/Georgia/W22-750A/2022	black vulture
OQ584705	8 (NS)	USA	A/black vulture/Virginia/W22-667C/2022	black vulture
OQ584713	8 (NS)	USA	A/black vulture/Georgia/W22-749A/2022	black vulture
OQ584721	8 (NS)	USA	A/black vulture/Georgia/W22-675A/2022	black vulture
OQ584729	8 (NS)	USA	A/black vulture/Georgia/W22-404B/2022	black vulture
OQ584737	8 (NS)	USA	A/black vulture/Georgia/W22-395/2022	black vulture
OQ584745	8 (NS)	USA	A/black vulture/Virginia/W22-662A/2022	black vulture
OQ584795	8 (NS)	USA	A/black vulture/Georgia/W22-719C/2022	black vulture
OQ632902	8 (NS)	France	A/chicken/France/21328/2021	chicken
OQ683462	8 (NS)	Colombia	A/chicken/Colombia/Bolivar/3500/2022	chicken
OQ683470	8 (NS)	Colombia	A/chicken/Colombia/Choco/3502/2022	chicken
OQ683478	8 (NS)	Colombia	A/chicken/Colombia/Choco/3504/2022	chicken
OQ683486	8 (NS)	Colombia	A/chicken/Colombia/Cordoba/3499/2022	chicken
OQ683494	8 (NS)	Colombia	A/chicken/Colombia/Magdalena/3503/2022	chicken
OQ683502	8 (NS)	Colombia	A/wild duck/Colombia/Choco/3501/2022	wild duck
OQ694810	8 (NS)	USA	A/black vulture/Georgia/W22-1048/2022	black vulture
OQ694850	8 (NS)	USA	A/snow goose/Kansas/W22-1143/2022	snow goose
OQ694858	8 (NS)	USA	A/black vulture/North Carolina/W22-1078/2022	black vulture
OQ694866	8 (NS)	USA	A/black vulture/South Carolina/W22-1112/2022	black vulture
OQ694874	8 (NS)	USA	A/black vulture/South Carolina/W22-1080B/2022	black vulture
OQ694882	8 (NS)	USA	A/snow goose/Louisana/W22-1163/2022	snow goose
OQ694890	8 (NS)	USA	A/black vulture/Georgia/W22-1049/2022	black vulture
OQ694898	8 (NS)	USA	A/black vulture/Georgia/W22-1057B/2022	black vulture
OQ694906	8 (NS)	USA	A/Canada goose/North Carolina/W22-1055/2022	Canada goose
OQ694916	8 (NS)	USA	A/black vulture/Georgia/W22-1057D/2022	black vulture
OQ694924	8 (NS)	USA	A/mallard/North Carolina/W22-1114A/2022	mallard
OQ694932	8 (NS)	USA	A/snow goose/Louisana/W22-1146A/2022	snow goose
OQ694940	8 (NS)	USA	A/black vulture/North Carolina/W22-1051A/2022	black vulture
OQ694948	8 (NS)	USA	A/black vulture/Tennessee/W22-1150B/2022	black vulture
OQ694956	8 (NS)	USA	A/black vulture/Tennessee/W22-1149/2022	black vulture
OQ696056	8 (NS)	USA	A/Ross's goose/Kansas/W22-1154/2022	Ross's goose
OQ696067	8 (NS)	USA	A/red-tailed hawk/Kansas/W22-1155/2022	red-tailed hawk
OQ732944	8 (NS)	USA	A/black vulture/Georgia/W22-969B/2022	black vulture
OQ732952	8 (NS)	USA	A/black vulture/South Carolina/W22-1080A/2022	black vulture

OQ732960	8 (NS)	USA	A/blue-winged teal/Minnesota/AV22-690/2022	blue-winged teal
OQ732968	8 (NS)	USA	A/red-tailed hawk/Kentucky/W23-143/2022	red-tailed hawk
OQ732976	8 (NS)	USA	A/blue-winged teal/Texas/UGAI22-2966/2022	blue-winged teal
OQ732984	8 (NS)	USA	A/black vulture/Georgia/W22-933C/2022	black vulture
OQ732992	8 (NS)	USA	A/bald eagle/North Carolina/W23-142B/2023	bald eagle
OQ733000	8 (NS)	USA	A/blue-winged teal/Texas/UGAI22-3268/2022	blue-winged teal
OQ733008	8 (NS)	USA	A/blue-winged teal/Minnesota/UGAI22-3620/2022	blue-winged teal
OQ733016	8 (NS)	USA	A/blue-winged teal/Texas/UGAI22-3250/2022	blue-winged teal
OQ733024	8 (NS)	USA	A/bald eagle/North Carolina/W23-142A/2023	bald eagle
OQ733032	8 (NS)	USA	A/blue-winged teal/Texas/UGAI22-3189/2022	blue-winged teal
OQ733040	8 (NS)	USA	A/blue-winged teal/Minnesota/AV22-675/2022	blue-winged teal
OQ733048	8 (NS)	USA	A/black vulture/North Carolina/W22-1079C/2022	black vulture
OQ733056	8 (NS)	USA	A/black vulture/Louisiana/W23-119/2023	black vulture
OQ733064	8 (NS)	USA	A/black vulture/Georgia/W22-972A/2022	black vulture
OQ733072	8 (NS)	USA	A/bald eagle/South Carolina/W23-142A/2023	bald eagle
OQ733080	8 (NS)	USA	A/blue-winged teal/Texas/UGAI22-3226/2022	blue-winged teal
OQ733096	8 (NS)	USA	A/bald eagle/Virginia/W22-101/2023	bald eagle
OQ733104	8 (NS)	USA	A/blue-winged teal/Texas/UGAI22-3190/2022	blue-winged teal
OQ733112	8 (NS)	USA	A/blue-winged teal/Texas/UGAI22-2961/2022	blue-winged teal
OQ733120	8 (NS)	USA	A/black vulture/Georgia/W22-1056A/2022	black vulture
OQ733128	8 (NS)	USA	A/blue-winged teal/Minnesota/UGAI22-3611/2022	blue-winged teal
OQ733136	8 (NS)	USA	A/black vulture/Louisiana/W23-118/2023	black vulture
OQ733144	8 (NS)	USA	A/Canada goose/Virginia/W22-773/2022	Canada goose
OQ733152	8 (NS)	USA	A/blue-winged teal/Minnesota/AV22-680/2022	blue-winged teal
OQ733160	8 (NS)	USA	A/mallard duck/Minnesota/AV22-632/2022	mallard duck
OQ734882	8 (NS)	USA	A/red-tailed hawk/Kansas/W22-1226/2022	red-tailed hawk
OQ734890	8 (NS)	USA	A/bald eagle/Tennessee/W23-003/2022	bald eagle
OQ734898	8 (NS)	USA	A/American green-winged teal/Texas/UGAI22-3462/2022	American green-winged teal
OQ734906	8 (NS)	USA	A/Cooper's hawk/Kansas/W22-1206/2022	Cooper's hawk
OQ734922	8 (NS)	USA	A/brown pelican/North Carolina/W23-019/2022	brown pelican
OQ734930	8 (NS)	USA	A/double-crested cormorant/North Carolina/W22-1215C/2022	double-crested cormorant
OQ734941	8 (NS)	USA	A/herring gull/North Carolina/W1215B/2022	herring gull
OQ734968	8 (NS)	USA	A/black vulture/North Carolina/W22-1213/2022	black vulture
OQ734976	8 (NS)	USA	A/bald eagle/Virginia/W22-1222/2022	bald eagle
OQ737757	8 (NS)	USA	A/black vulture/Georgia/W22-719B/2022	black vulture
OQ737765	8 (NS)	USA	A/blue-winged teal/Minnesota/UGAI22-3591/2022	blue-winged teal
OQ737773	8 (NS)	USA	A/great black-backed gull/North Carolina/W22-1215A/2022	great black-backed gull
OQ747884	8 (NS)	Peru	A/Peruvian_pelican/Peru/A074/2022	Peruvian_pelican
OQ747885	8 (NS)	Peru	A/Belcher's_gull/Peru/A102/2022	Belcher's_gull
OQ747886	8 (NS)	Peru	A/Peruvian_pelican/Peru/A106/2022	Peruvian_pelican
OQ747887	8 (NS)	Peru	A/Belcher's_gull/Peru/A267/2022	Belcher's_gull
OQ747888	8 (NS)	Peru	A/American_kestrel/Peru/A273/2022	American_kestrel
OQ789279	8 (NS)	USA	A/American wigeon/South Carolina/AH0195145/2021	American wigeon
OQ851651	8 (NS)	USA	A/Pekin duck/California/T2202390/2022	Pekin duck
OQ954524	8 (NS)	USA	A/bald eagle/South Carolina/W23-201A/2023	bald eagle
OQ954532	8 (NS)	USA	A/bald eagle/South Carolina/W23-201B/2023	bald eagle
OQ954540	8 (NS)	USA	A/black vulture/Louisiana/W23-166/2023	black vulture
OQ982392	8 (NS)	USA	A/mallard duck/Minnesota/UGAI22-3846/2022	mallard duck
OQ982400	8 (NS)	USA	A/bald eagle/North Carolina/W23-012/2022	bald eagle
OQ982408	8 (NS)	USA	A/blue-winged teal/Louisiana/UGAI22-3889/2022	blue-winged teal
OQ982416	8 (NS)	USA	A/blue-winged teal/Minnesota/UGAI22-3834/2022	blue-winged teal
OQ982424	8 (NS)	USA	A/blue-winged teal/Louisiana/UGAI22-3875/2022	blue-winged teal
