# Supplementary material for: Spreading of the High-Pathogenicity Avian Influenza (H5N1) Virus of Clade 2.3.4.4b into Uruguay
Source: Viruses. 2023 Sep 11;15(9):1906. doi: 10.3390/v15091906 (PMC10536905; doi:10.3390/v15091906)
Supplement: Supplementary file 1 [file viruses-15-01906-s001.zip › viruses-2590692-supplementary.pdf]

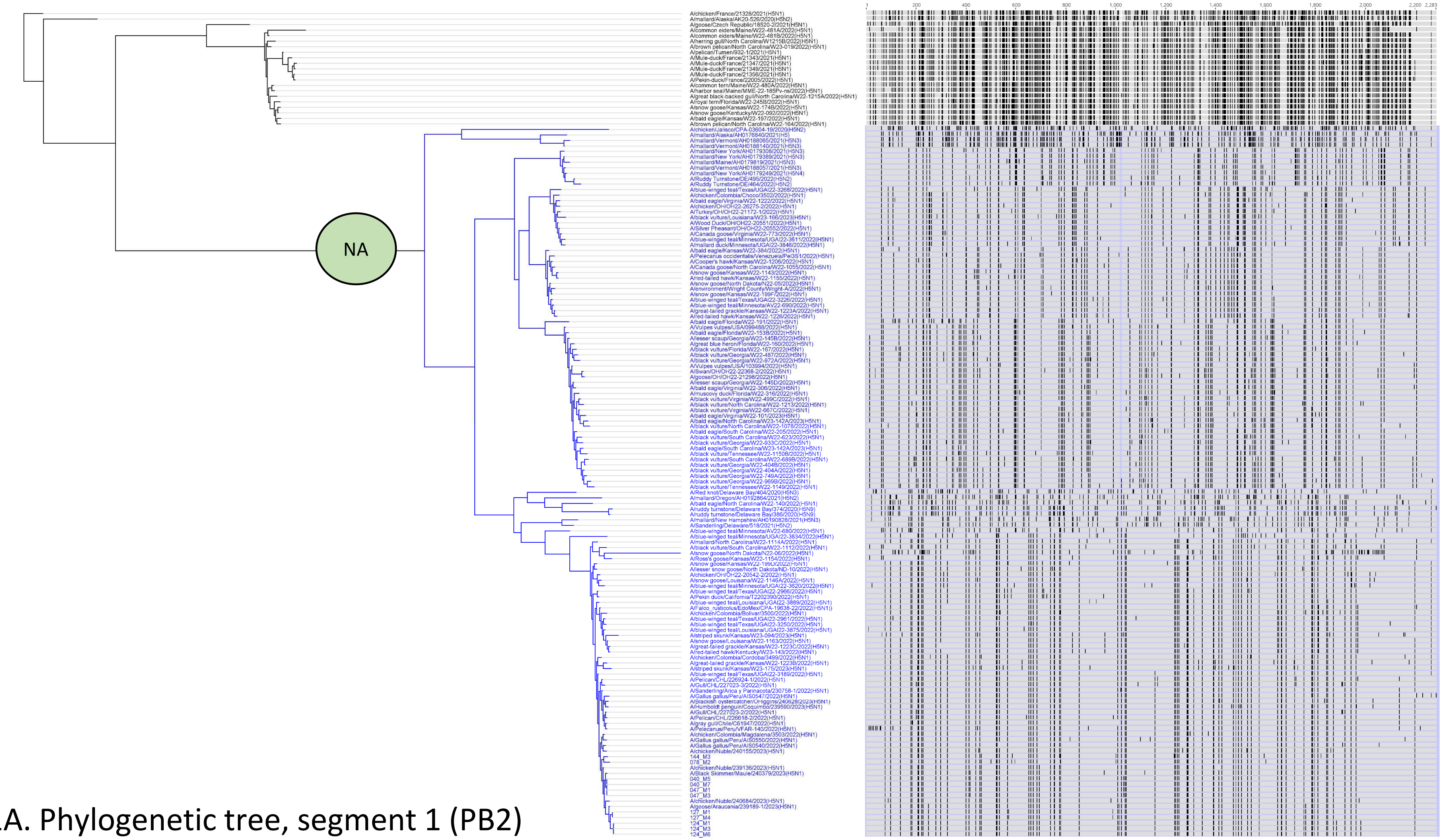

Figure S1A. Phylogenetic tree, segment 1 (PB2)

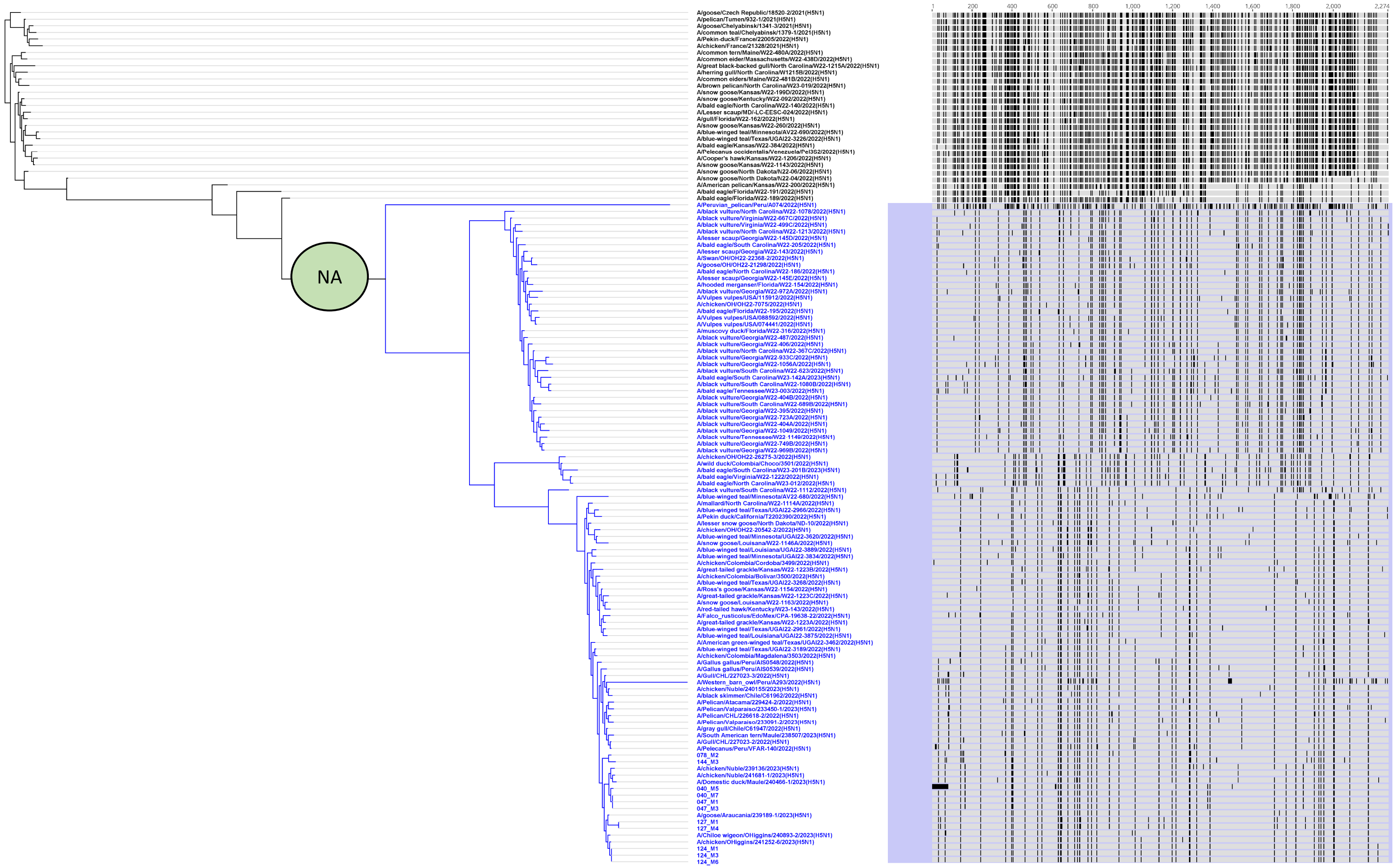

Figure S1B. Phylogenetic tree, segment 2 (PB1)

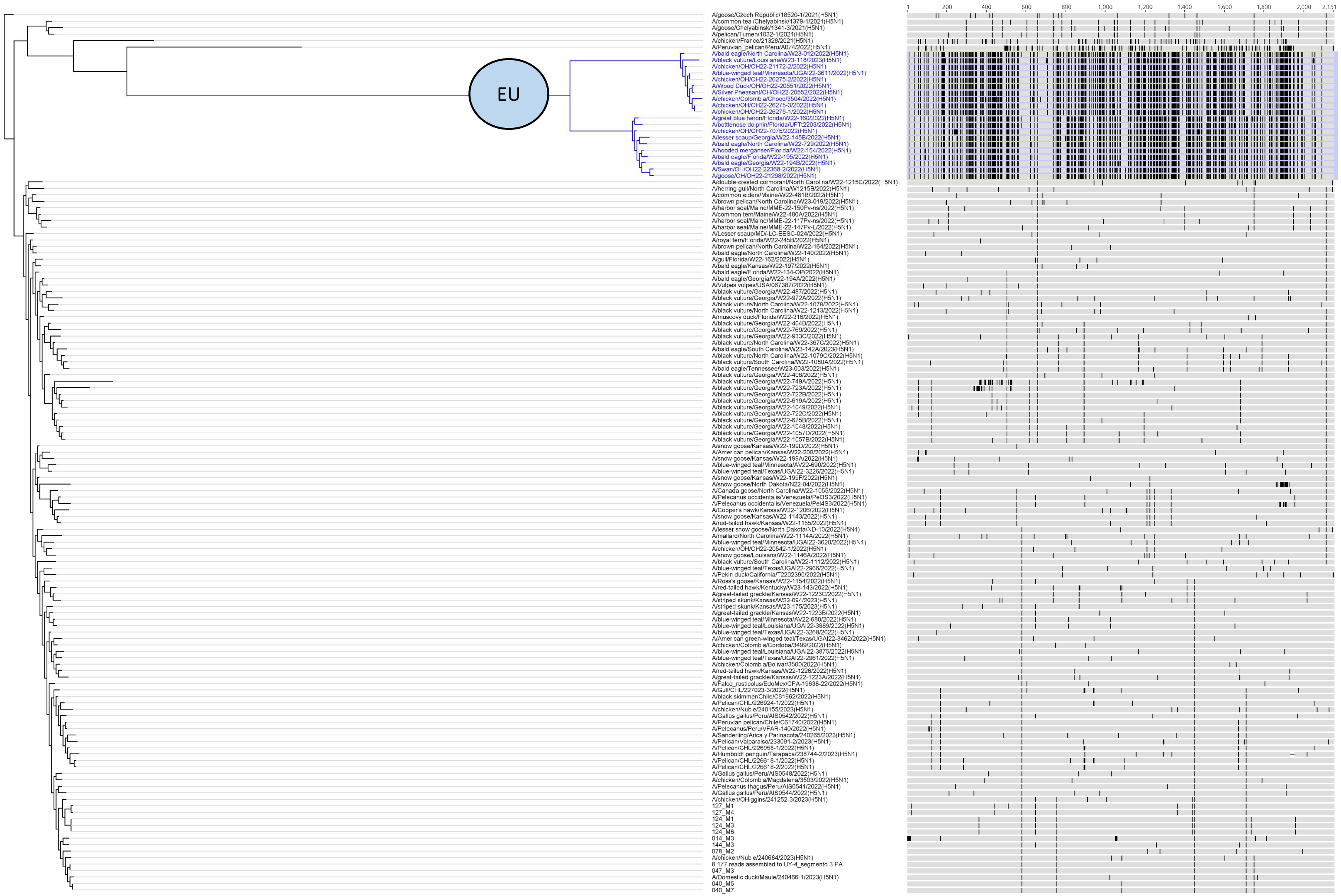

Figure S1C. Phylogenetic tree, segment 3 (PA)

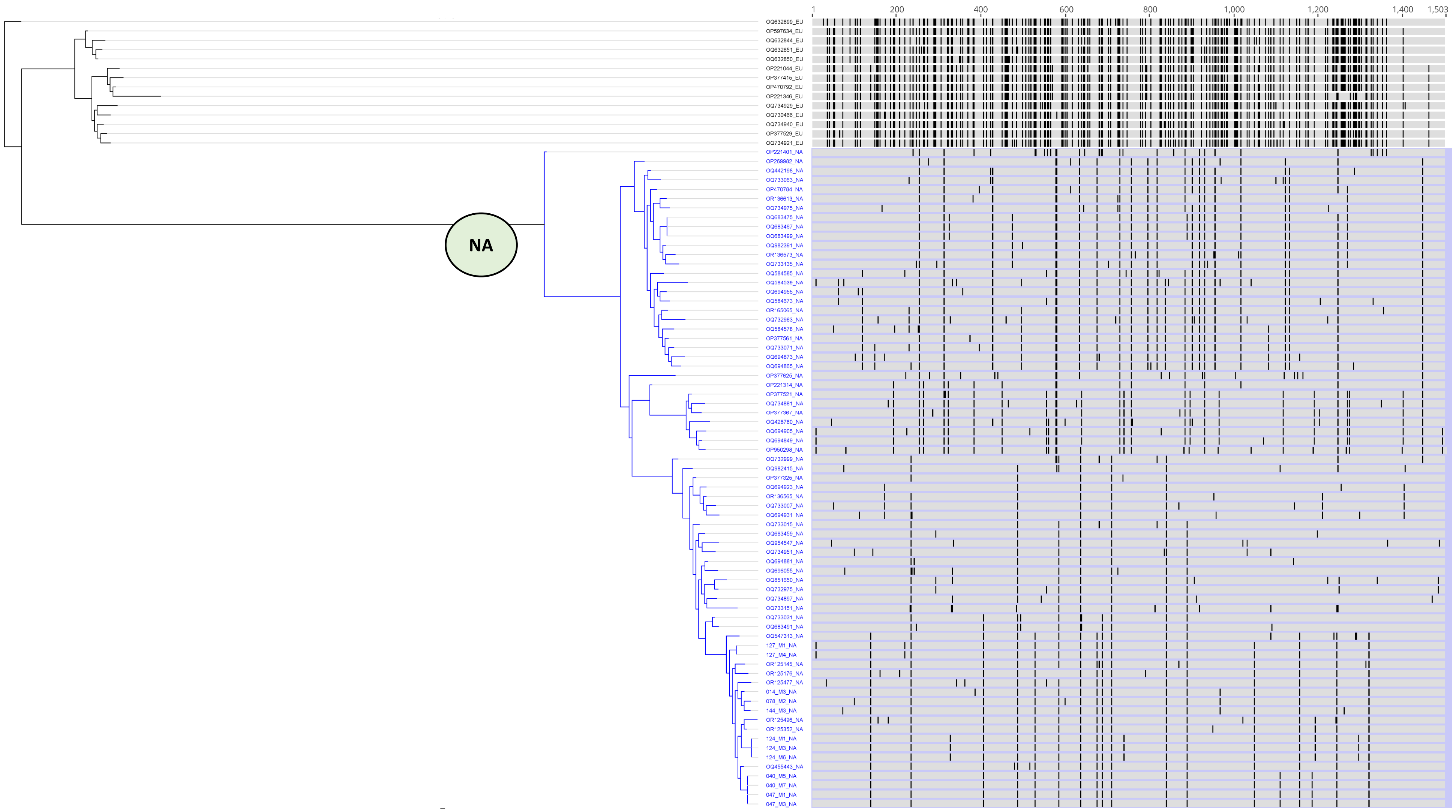

Figure S1D. Phylogenetic tree, segment 5 (NP)

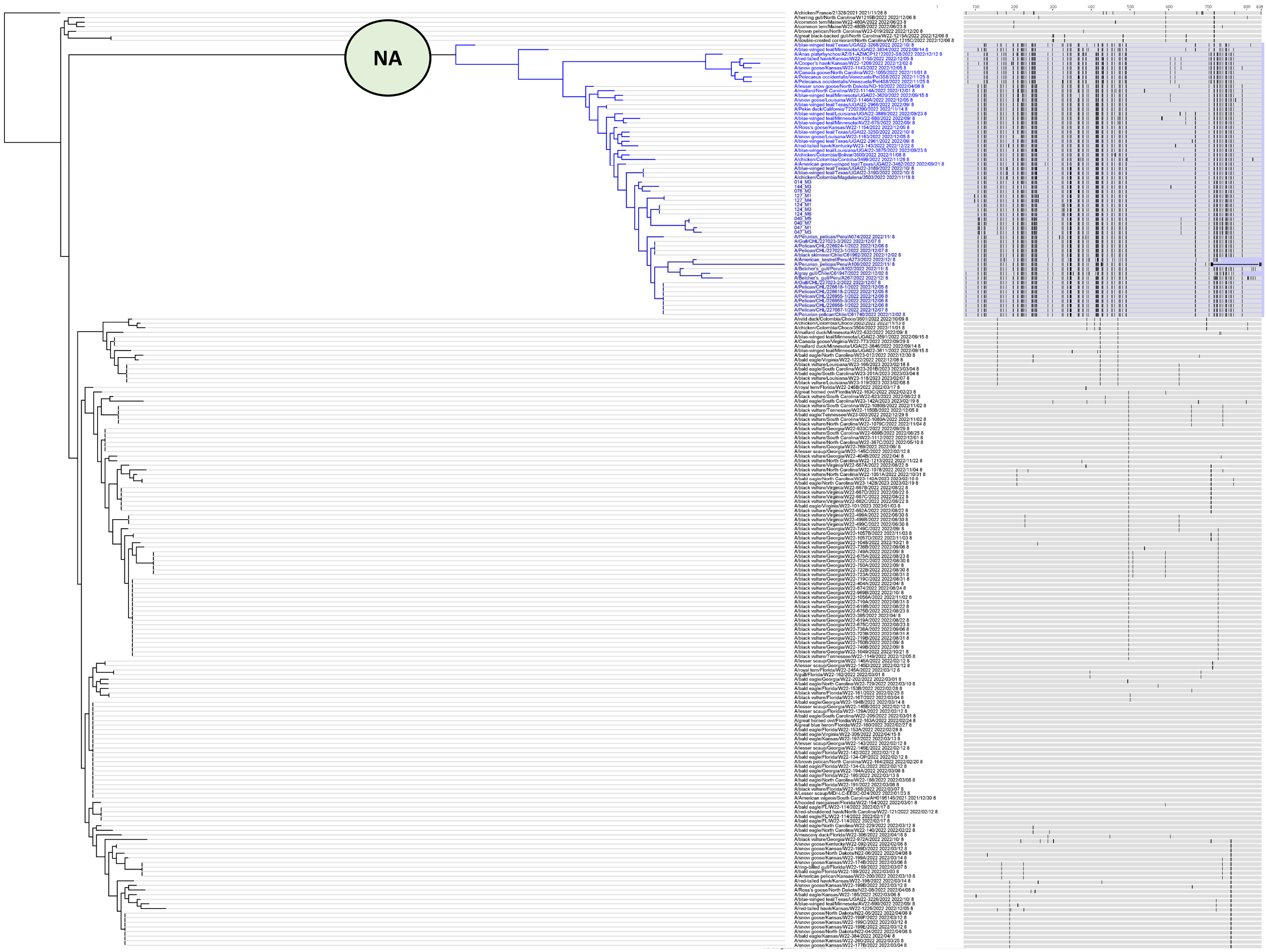

Figure S1E. Phylogenetic tree, segment 8 (NS)
